# Sex differences in pain perception and modulation in the brain: effects of insular cortex stimulation on chronic pain relief

**DOI:** 10.1093/braincomms/fcaf362

**Published:** 2025-09-17

**Authors:** Minjee Kwon, Kyeongmin Kim, Young-Ji Eum, Guanghai Nan, Leejeong Kim, Hyeji Park, Un Jeng Kim, Jin-Hun Sohn, Chaejoon Cheong, Jee-Hyun Cho, Myeounghoon Cha, Bae Hwan Lee

**Affiliations:** Department of Nursing, Kyungil University, Gyeongsan 38428, Republic of Korea; Department of Physiology, Yonsei University College of Medicine, Seoul 03722, Republic of Korea; Graduate School of Medical Science, Brain Korea 21 Project, Yonsei University College of Medicine, Seoul 03722, Republic of Korea; Biopharmaceutical Research Center, Korea Basic Science Institute, Cheongju 28119, Republic of Korea; Department of Physiology, Yonsei University College of Medicine, Seoul 03722, Republic of Korea; Graduate School of Medical Science, Brain Korea 21 Project, Yonsei University College of Medicine, Seoul 03722, Republic of Korea; Department of Physiology, Yonsei University College of Medicine, Seoul 03722, Republic of Korea; Graduate School of Medical Science, Brain Korea 21 Project, Yonsei University College of Medicine, Seoul 03722, Republic of Korea; Department of Physiology, Yonsei University College of Medicine, Seoul 03722, Republic of Korea; Department of Physiology, Yonsei University College of Medicine, Seoul 03722, Republic of Korea; Department of Physiology, Yonsei University College of Medicine, Seoul 03722, Republic of Korea; Center for Bio-Imaging and Translational Research, Korea Basic Science Institute, Cheongju 28119, Republic of Korea; Biopharmaceutical Research Center, Korea Basic Science Institute, Cheongju 28119, Republic of Korea; Department of Physiology, Yonsei University College of Medicine, Seoul 03722, Republic of Korea; Department of Physiology, College of Medicine, Soonchunhyang University, Cheonan 31151, Republic of Korea; Department of Physiology, Yonsei University College of Medicine, Seoul 03722, Republic of Korea; Graduate School of Medical Science, Brain Korea 21 Project, Yonsei University College of Medicine, Seoul 03722, Republic of Korea; Brain Research Institute, Yonsei University College of Medicine, Seoul 03722, Republic of Korea

**Keywords:** brain network plasticity, insular cortex stimulation, affective pain processing, sex as a biological variable, sex-specific therapeutics

## Abstract

Sex-specific differences in brain activation related to chronic pain remain poorly understood. In particular, how stimulation of the insular cortex—a key modulator of pain processing—differentially affects neural pathways in males and females is not well characterized. This study aimed to determine whether insular cortex stimulation activates distinct pain modulation circuits in a sex-dependent manner using a rat model of chronic pain. Understanding these differences may help inform more personalized and effective pain treatment. Neuropathic pain was induced in male and female rats to establish a chronic pain model, followed by insular cortex stimulation. Pain sensitivity was assessed using mechanical allodynia tests to evaluate the behavioural responses. Functional brain connectivity was examined using diffusion tensor imaging, and fractional anisotropy values were calculated across key brain regions. Correlation analyses were conducted between behavioural pain scores and fractional anisotropy values to investigate the relationship between the structural connectivity changes and pain modulation. Under sham conditions, males exhibited lower fractional anisotropy values than females. In the pain condition, both sexes showed reduced fractional anisotropy values; however, females displayed a significantly greater decrease in the ventral posterior thalamic nucleus–amygdala pathway than did males. Following insular cortex stimulation, males showed a more pronounced increase in fractional anisotropy values, with significant sex differences observed in the ventral posterior thalamic nucleus–anterior cingulate cortex, ventral posterior thalamic nucleus–insular cortex, ventral posterior thalamic nucleus–nucleus accumbens, ventral posterior thalamic nucleus–primary somatosensory cortex, primary somatosensory cortex–insular cortex and primary somatosensory cortex–prefrontal cortex pathways. These findings underscore the sex-related differences in brain activation and pain modulation pathways in chronic pain. A deeper understanding of these mechanisms may inform the development of more effective sex-tailored interventions for chronic pain and improve clinical outcomes.

## Teaser

Kwon *et al*. reported that neuropathic pain alters structural brain connectivity in rats, as measured by diffusion tensor imaging. Intermittent cortical stimulation partially restored white matter integrity, particularly in females. These findings suggest a sex-dependent mechanism in pain-related brain network reorganization and the potential for neuromodulatory intervention.

## Graphical Abstract

**Graphical Abstract:**
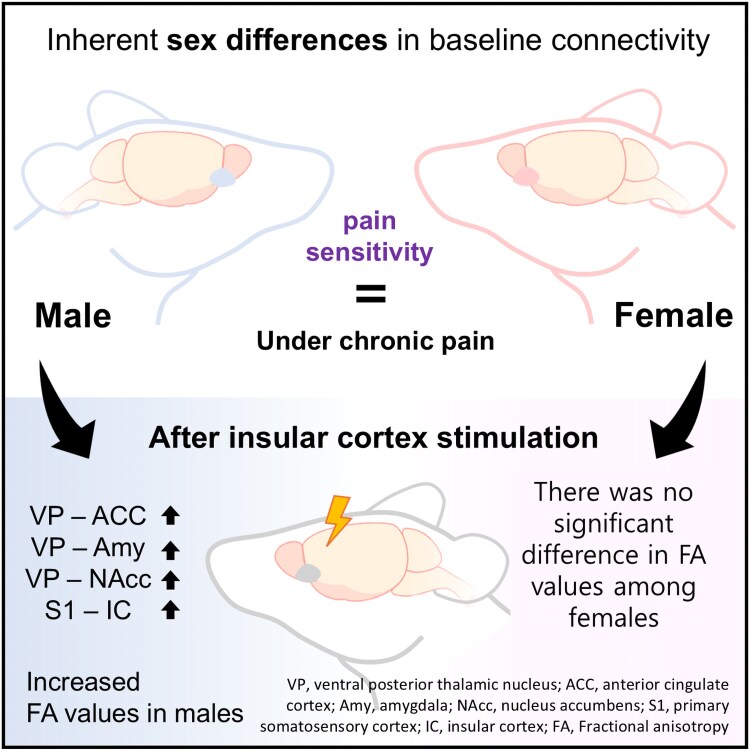

## Introduction

Research on sex differences in pain began in the late 1990s and has since expanded to a wide range of experimental and clinical studies.^1,2^ Recent advances in non-invasive brain research methods have provided a wealth of opportunities for analysing the brain's fine structures, activities and connections. Recent studies have suggested that females may experience pain more severely than males, regardless of the pain type (e.g. cancer, musculoskeletal, or post-surgical pain) or measurement method (e.g. pain threshold, tolerance and intensity rating).^3^ The reasons for these variations are not yet fully understood; however, several clinical and preclinical trials are ongoing to gain further insights.^4,5^ Additionally, identifying the factors that contribute to women experiencing greater pain levels in chronic pain conditions, such as neuropathic, inflammatory and other types of pain, is a key focus in the field of sex-specific and patient-tailored therapies.^6,7^ Understanding the differences between males and females in pain neurobiology is crucial for effective pain treatment. Previous studies have shown that neuroimmune interactions in the dorsal horn of the spine play a significant role in sex differences in pain, with testosterone in males and T-cell changes in females being important factors.^8,9^

Researchers have recently attempted to establish a correlation between sexual dimorphism in the brain and pain responses.^10-13^ However, there are various methods for analysing brain function, and individual variation exists even within populations of the same sex, depending on the measurement method used.^14^ Advances continue to be made in non-invasive neuroimaging techniques, such as CT, PET and functional magnetic resonance imaging (fMRI). These techniques provide an important means of better understanding sex-related differences in the brain.^15^ Sexual dimorphism in the brain has been demonstrated in CT studies, which revealed differences between the sexes in the reduction of regional cerebral blood flow with increasing age^16^; these differences can lead to brain atrophy.^17^ PET studies analysing glucose metabolism in the brain have revealed higher glucose metabolism in the hypothalamus in females than in males.^18^ One study demonstrated age-related changes in the entorhinal and parahippocampal volumes only in males, indicating that age-related changes in the brain are sex-specific.^19^ In addition, MRI studies have demonstrated structural and functional sex differences in grey matter volume, regional homogeneity and functional connectivity in distinct ways. These structural and functional differences between the sexes provide a foundation for understanding the intricate relationships between brain structure and function, and the complexity of sex differences in behaviour.^20,21^

Diffusion tensor imaging (DTI) is an MR-based imaging technique used to characterize the microstructures of large white matter tracts under physiological and pathological conditions.^22^ DTI is a useful method for assessing a range of neurological changes in the brain that cannot be assessed by MRI. DTI can be used to determine the direction of movement of water molecules in the tissue and evaluate the anisotropic properties of brain connections. The fractional anisotropy (FA) value, which represents the proportion of anisotropic components in the total diffusion tensor, indirectly indicates brain fibre bundle integrity.^23-25^

The insular cortex (IC) links sensory pain and limbic system networks.^26,27^ Of all the brain regions involved in pain processing, only the IC and secondary somatosensory cortex (S2) produce pain when stimulated.^28^ The IC correlates with brain structures in the descending pain pathway from the periaqueductal grey (PAG) to the spinal cord through the rostral ventromedial medulla.^29^ Furthermore, the IC is frequently activated by noxious pain stimuli,^26,30^ and its activity changes with the aggregation of chronic pain.^31^ Since the IC integrates sensory, emotional and cognitive processes and is involved in aversive and motivational salience, persistent alterations in its circuitry and synaptic physiology may underlie the emergence of chronic pain and its associated complications.^32^ Our previous findings are aligned with those of previous studies showing reduced pain sensations with the application of electrical stimulation to the IC.^30,33,34^ These data are consistent with those of other studies showing that the painful sensation was reduced when neuronal modulation was applied to the IC.^35^ However, few studies have demonstrated the pain attenuation effects of insular cortex stimulation (ICS),^26,36^ and none have compared pain perception and brain connectivity between sexes after ICS administration.

This study aimed to analyse brain functional connectivity and investigate sexual dimorphism using FA values in rats with chronic pain. We also aimed to confirm the previously reported pain-relieving effect of ICS^30^ and compare brain microstructural organization and differences between male and female rats. In the present study, all references to sex differences refer strictly to biological sex, as determined by the gonadal phenotype at birth, and behavioural and neuroanatomical comparisons were conducted between biologically defined male and female groups.

## Materials and methods

### Animals

Male and female Sprague Dawley rats (240 ± 10 g; Harlan, Koatec, Pyeongtaek, Korea) were used in all the experiments. A total of 57 Sprague–Dawley rats (27 males and 30 females) were randomly assigned to three experimental groups: Sham (male *n* = 9; female *n* = 10), NP (nerve injury; male *n* = 9; female *n* = 10), and ICS (insular cortex stimulation; male *n* = 9; female *n* = 10). The sample sizes were determined based on effect size estimates from previous studies and confirmed through post hoc power analysis. Assuming a medium-to-large effect size (Cohen’s d = 0.8–1.2), group sizes of *n* = 9–10 per sex per condition achieved statistical power of >0.80 at α = 0.05 for detecting group- and sex-related differences in behavioural outcomes. Animals were individually housed under controlled conditions (22 ± 2°C, 50–60% humidity, 12 h light-dark cycle) with food and water available ad libitum. All procedures were conducted in accordance with the National Institutes of Health guidelines and approved by the Institutional Animal Care and Use Committee (IACUC) of the Yonsei University Health System (approval no. 2019-0225).

### Electrode implantation and neuropathic surgery

Animals were randomly assigned to one of three experimental groups: Sham group, which underwent electrode implantation without nerve injury or stimulation; NP group, which received peripheral nerve injury and electrode implantation without stimulation; and ICS group, which received nerve injury followed by electrical stimulation of the IC. All surgical procedures were performed under sterile conditions. General anaesthesia was induced with pentobarbital sodium (50 mg/kg, intraperitoneally [i.p.]), preceded by atropine premedication (5 mg/kg, i.p.) to prevent the secretion of mucus. Deeply anaesthetized rats were placed in a stereotaxic frame. A stimulation electrode (MS308, P1 Technologies, Roanoke, VA, USA) was unilaterally implanted into the right rostral insular cortex (IC; 1.0 mm anterior to bregma, 5.0 mm lateral to midline and 7.7 mm below the skull surface) of all animals in this experiment. Animals were allowed to recover for three days following electrode implantation.^30^ Neuropathic surgery was performed as described previously.^37^ Briefly, the rats were anaesthetized with 5% isoflurane in an induction chamber. Following a longitudinal incision of the skin and muscles in the left lateral thigh, the tibial and sural nerves were tightly ligated with 5-0 black silk and subsequently transected, while the common peroneal nerve was not transected. In the sham group, rats underwent the same surgical procedure without any nerve injury.

### Mechanical threshold measurement

Behavioural tests were conducted to compare the mechanical withdrawal thresholds (MWT) of male and female rats. The MWT was measured over 14 days using an electronic von Frey (No. 38450, Ugo Basile, Varese, Italy). The rats were placed individually in acrylic cages on a wire mesh and allowed to habituate for 15 min. The test was repeated seven times for each rat. The average values were calculated, except for the minimum and maximum values of the data. Male and female rats underwent behavioural testing at separate times. Before each measurement, the acrylic cage was wiped with 70% ethanol. A researcher blinded to the experimental group conducted all behavioural tests.

### Brain stimulation of insular cortex

ICS was performed as described previously.^30^ Briefly, the electrode was connected to a stimulator (A385, WPI, Sarasota, FL, USA) for repetitive ICS. Repetitive ICS was performed 30 min once a day from postoperative day (POD) 7 to POD 14. The stimulation frequency and intensity of ICS were set to 50 Hz and 12 μA, respectively, based on our prior experimental results.^30^ The pulse width was set to 200 μs. In the Sham and NP groups, the electrode was connected to a stimulator, but no electrical stimulation was provided; this was called ‘sham-ICS.’

### Animal preparation for DTI MRI-imaging

Prior to animal MR imaging and mounting procedure, rats were euthanized and perfused with phosphate-buffered saline (PBS, pH 7.4), followed by 4% paraformaldehyde (PFA) in PBS. The brains were subsequently extracted and post-fixed in 4% PFA for 24 h. Afterward, the brains were rinsed in PBS and stored at 4°C in Fluorinert (FC-770, Sigma, St. Louis, MI, US) until DTI analysis. Prior to MRI scanning, the brains were placed inside 10 mL syringes (Fisher Scientific, Hampton, NH, US) with an outer diameter of approximately 1.6 cm and a length of 2 cm from the cap to the tip. Each brain was then immersed in liquid fluoride and fixed without agitation using a syringe to avoid air bubbles. The tip of the syringe was secured using silicone.

### MRI data acquisition

All ex vivo rat brain MR imaging was performed using a Bruker Biospec 9.4 T/20 cm horizontal bore magnet (Bruker, BioSpin, Ettlingen, Germany) with a 1H 35 mm circularly polarized transmit/receive volume coil. T2-weighted images were acquired with a TE of 26 ms, a field of view (FOV) of 2.56 × 2.56 cm² and an isotropic resolution of 0.100 cm. Diffusion tensor data were acquired using a spin-echo-echo planar imaging (SE-EPI) pulse sequence. For DTI acquisition, the acquisition parameters were TR = 3 s, TE = 50 ms, slice thickness of 0.5 mm, a field of view of 2.56 × 2.56 cm2, a b-factor of 3000 s/mm2, a diffusion pulse width of 4 ms and interpulse duration of 20 ms, and the voxel size of 0.134 mm × 0.134 mm × 0.500 mm = 0.009 mm³. An automatic quality control routine was used to check the b-table for accuracy, and the diffusion tensor was calculated as described previously.^38^

### Region of interest selection and image processing

Tractography was used to select nine brain regions associated with pain processing as ROIs, including the anterior cingulate cortex (ACC), prefrontal cortex (PFC), IC, primary somatosensory cortex (S1), secondary somatosensory cortex (S2), ventral posterior thalamic nucleus (VP), PAG, nucleus accumbens (NAcc) and amygdala (Amy). The high-resolution SIGMA rat brain template^39^ and Paxinos and Watson atlas were used.^40^ ROI masks were obtained from the SIGMA atlas using the Atlas Normalization Toolbox and Elastix 2 (ANTx2, University Medicine Berlin, Berlin, Germany). The DTI data were processed using ANTx2, the Functional Magnetic Resonance Imaging of the Brain (FMRIB) software library version 6.0.2 (FSL, created by the Analysis Group, FMRIB, Oxford, UK), and MRtrix3 (https://www.mrtrix.org).^41^ The ANTx2 tool was used to perform format conversion to Neuroimaging Informatics Technology Initiative, re-orient to SIGMA space,^39^ and extract B0 images. Subsequently, all data were linearly registered and spatially normalized into the SIGMA space using FMRIB’s Linear Image Registration Tool function.^39^ The DTI data were denoised using MRtrix3 and corrected for distortions and motion artefacts using the eddy-correct tool in FSL.

### Tractography

DSI Studio was used to perform tractography analyses. Deterministic tractography was performed using the following global parameters: angular threshold of 60°, step size of 0.05 mm, minimum length of 1 mm, and termination if 600 000 seeds were reached. The tracking threshold was calculated using the DSI Studio to maximize the variance between the background and foreground. The maximum length was defined according to the anatomical distance between the two ROIs. Region of interest (ROI)-based tracking was employed to investigate the connectivity of brain regions associated with pain processing. Tracking resulted in the number of streamlines seeded on one ROI targeting the other ROI in the ipsilateral hemisphere.

### Statistical analysis

SPSS version 28 (IBM Corp., Armonk, NY, USA) was used for all statistical analysis. Pain sensitivity was defined as the mean value of the mechanical thresholds measured before and after stimulation for 14 days. Paired and unpaired *t*-tests were used to compare the data, with a significance threshold of *P* < 0.05. The mean FA values of the ROIs from the DTI analysis were visualized as heatmaps using the *heatmap.2* function of the ‘g plots’ package in R. The data were divided into male and female groups to generate one set of heatmaps, and the other set was created using the differences in FA values between the two groups. We also examined whether there was an association between FA values and pain sensitivity using Spearman’s correlation coefficient. All analyses were performed with sex as an independent biological variable, unless otherwise stated. All tests were two-tailed, and the threshold for significance was set at *P* < 0.05.

## Results

### Sex-dependent variations in pain behaviours and ICS-induced modulation of pain responses

**Figure 1:**
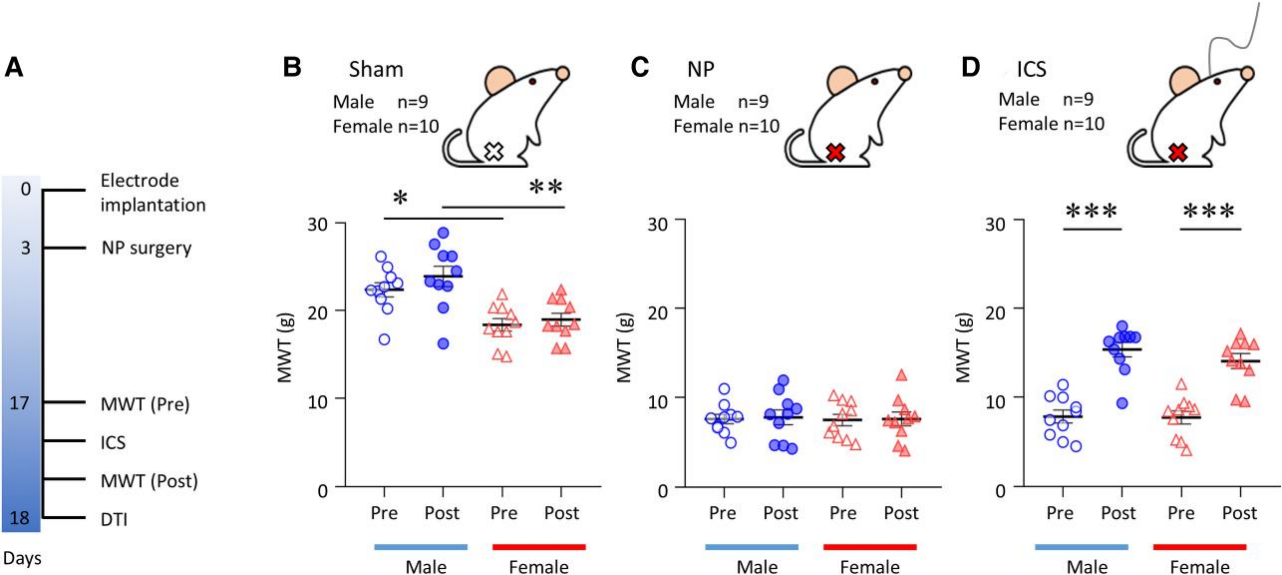
**Experimental timeline and comparison of mechanical threshold.** (**A**) The timeline illustrates the order of the surgical procedures, behavioural testing, and imaging assessments. (**B**) Comparison of mechanical withdrawal thresholds between male and female rats in the Sham group (no injury, no stimulation). (**C**) Mechanical withdrawal threshold comparison between male and female rats in the NP group (nerve injury without stimulation). (**D**) Mechanical withdrawal threshold comparison between male and female rats in the insular cortex stimulation group (nerve injury with insular stimulation). The white and red markers indicate the absence and presence of NP, respectively, while the line on the head represents insular cortex stimulation. Each data point in (**B**) to (**D**) represents an individual animal. Data are presented as mean ± SEM and analysed using one-way ANOVA followed by Tukey’s multiple comparisons test (*n*  *=* 27 males; *n*  *=* 30 females). Statistical significance between males and females is denoted by *P < 0.05, **P < 0.01. Significant differences between the pre- and post-treatment groups in the insular cortex stimulation group are indicated by ***P < 0.001.

Behavioural assessments revealed distinct pain responses across the three experimental conditions tested. Animals in the NP group (nerve injury without stimulation) displayed significant mechanical allodynia compared to those in the Sham group (no injury, no stimulation). In contrast, animals in the ICS group (nerve injury with insular stimulation) exhibited attenuated pain behaviour relative to the NP group. These effects were observed in both male and female rats, and statistical analyses were conducted with experimental conditions (Sham, NP and ICS) and sex (male and female) as between-subject factors. Figure 1 shows the MWT results before and after brain stimulation in the NP group. ‘Pre’ on the *x-*axis represents before brain stimulation, while ‘Post’ represents after brain stimulation.

In the Sham model, there was a significant difference between males and females in the MWT before and after (*P* < 0.05 (Male Pre versus Female Pre), *P* < 0.01 (Male Post versus Female Post)). However, no changes in the pain threshold between males and females were observed before and after sham-ICS (Male Pre 22.35 ± 0.82, Male Post 23.90 ± 1.16, Female Pre 18.37 ± 0.71 and Female Post 18.95 ± 0.73; Fig. 1B). Conversely, the pain threshold of males and females in the NP group dramatically decreased before and after sham-ICS compared with the Sham group. Within the NP group, as well as between the sexes, were not statistically significant (Male Pre 7.60 ± 0.52, Male Post 7.79 ± 0.83, Female Pre 8.03 ± 0.69 and Female Post 7.90 ± 0.80, *P* > 0.05, one-way ANOVA followed by Tukey's multiple comparison test) (Fig. 1C). In the ICS group, both male and female rats demonstrated a significant increase in mechanical pain threshold after receiving ICS (*P* < 0.001), whereas no significant differences between sexes were found (male Pre 7.84 ± 0.73, Male Post 15.15 ± 0.78, Female Pre 7.74 ± 0.72 and Female Post 14.06 ± 0.83, *P* < 0.05, one-way ANOVA with Tukey's multiple comparison test; Fig. 1D). For both male and female rats, the threshold did not recover to the Sham group level even after ICS. When comparing the degree of pain recovery, male rats exhibited a slightly higher level of recovery than female ones. These findings align with those of a previous study on chronic pain behaviour after ICS.^30^ Detailed MWT behavioural changes prior to DTI are shown in Supplementary Fig. 1.

### Neuroimaging analysis of ICS-induced brain changes in the context of chronic pain

To analyse the changes in brain functional connectivity between the groups, we compared the differences in DTI FA values across sex and groups. FA is a commonly used DTI metric because of its sensitivity in detecting damage to oriented structures, such as the white matter.^24,42^ FA values can be used to reconstruct the trajectories of white matter tracts corresponding to known neuroanatomy in three-dimensional space by calculating the orientation information from each voxel of the DTI through tractography. Higher FA values indicate more orderly movement of water molecules along tracts, whereas lower values indicate less well-connected tracts, which are more closely related to neural connections in the brain.^43^

**Figure 2:**
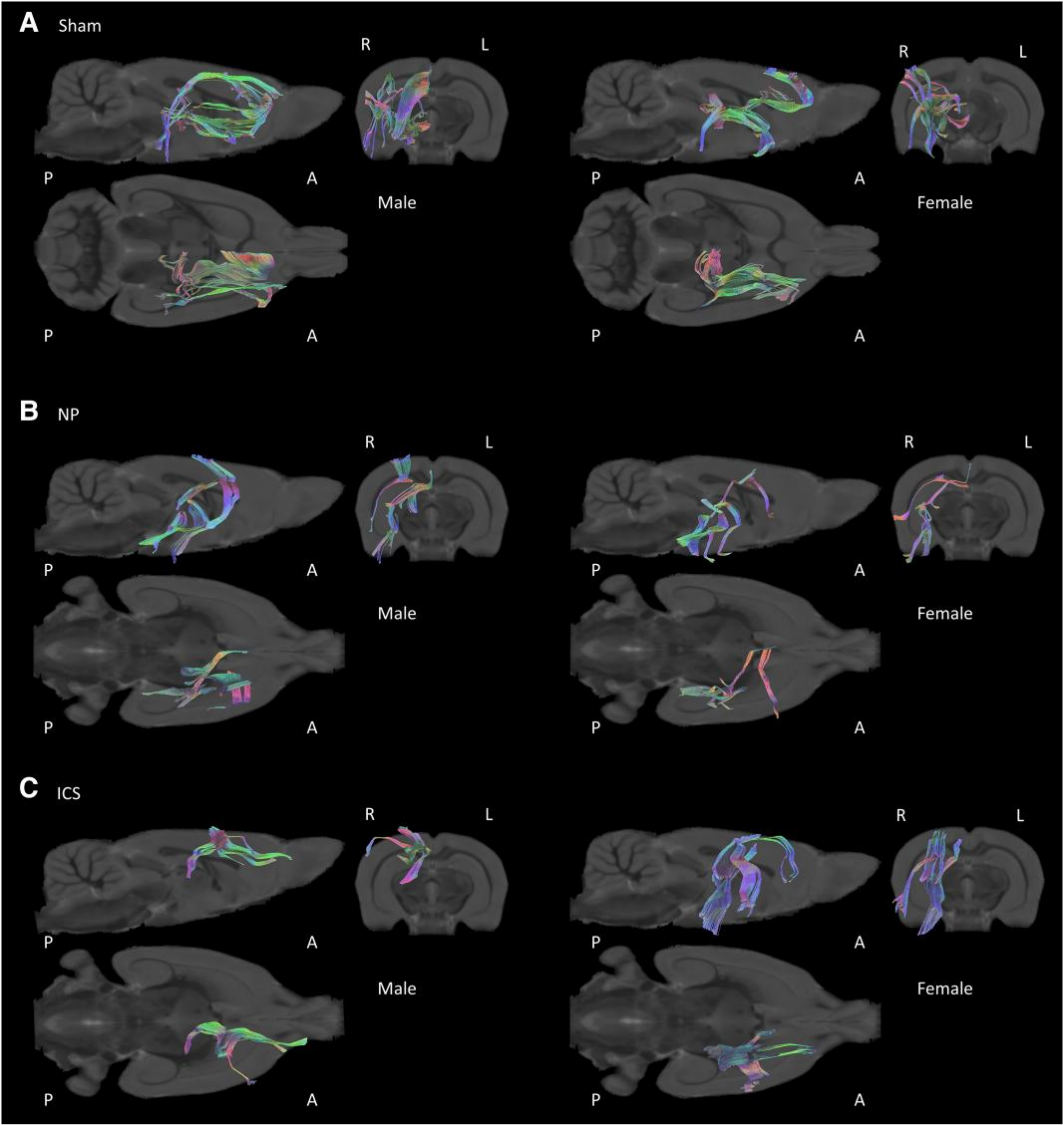
**Diffusion tensor imaging tractography.** Representative images of male and female rats in the (**A**) Sham, (**B**) NP and (**C**) insular cortex stimulation groups (**A–C**). Tract pathways are color-coded based on standard red-green-blue (RGB) encoding; red indicates right–left, blue indicates dorsal–ventral and green indicates anteroposterior orientations, reflecting the spatial directionality of terminal regions (A: anterior, P: posterior, L: left and R: right).

**Figure 3:**
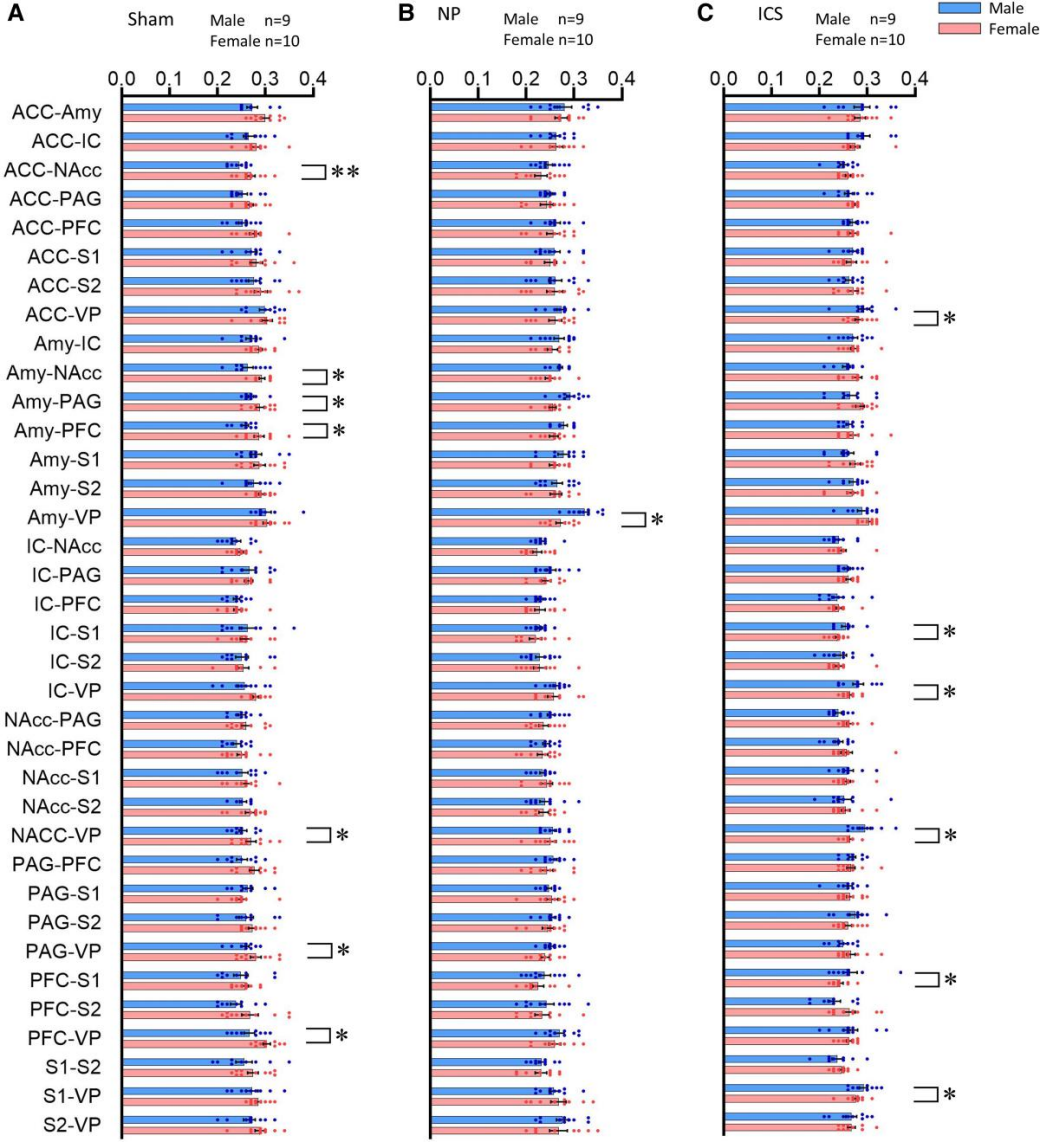
**Fractional anisotropy value comparisons across pain-related brain regions.** Fractional anisotropy values in functional connectivity between pain-related brain regions for sex differences in the (**A**) Sham, (**B**) NP and (**C**) insular cortex stimulation groups. Regions with statistically significant differences in FA values between males and females in each group are indicated. Unpaired t-tests were used for data comparisons, with statistical significance between males and females denoted as *P < 0.05 and **P < 0.01. Each data point represents an individual animal (*n*  *=* 27 males; *n*  *=* 30 females). ACC, anterior cingulate cortex; Amy, amygdala; IC, insular cortex; NAcc, nucleus accumbens; PAG, periaqueductal grey; PFC, prefrontal cortex; S1, primary somatosensory cortex; S2, secondary somatosensory cortex; VP, ventral posterior thalamic nucleus. Detailed data in Supplementary Tables 1 and 2.

**Figure 4:**
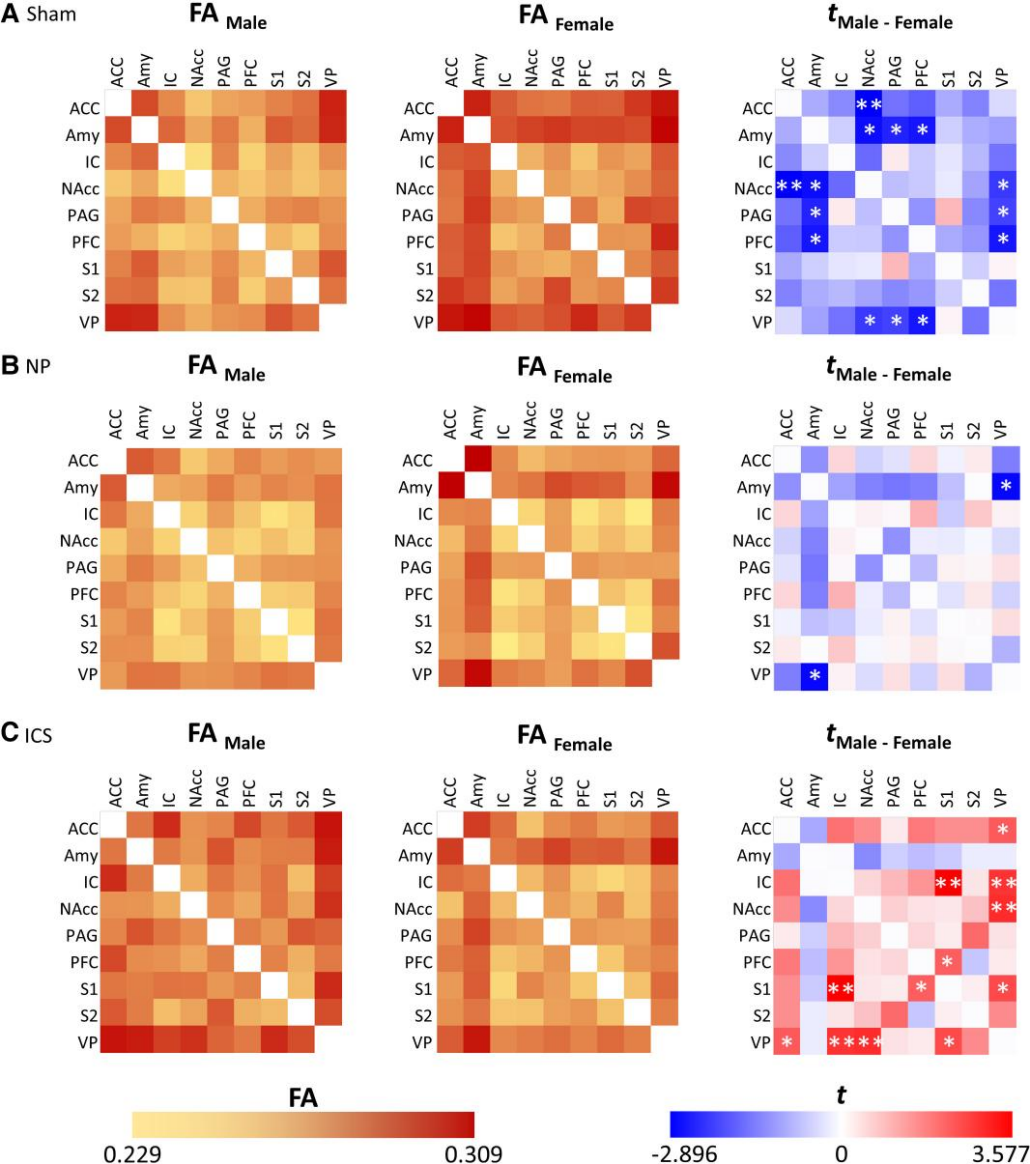
**Quantification of fractional anisotropy values for the sham, NP and insular cortex stimulation male/female groups.** Heatmap showing fractional anisotropy values in functional connectivity between pain-related brain regions for sex differences in the (**A**) Sham, (**B**) NP and (**C**) insular cortex stimulation groups. Error bars represent the SEM. The heatmaps on the left and middle column show the mean fractional anisotropy values of structural connectivity between two brain regions for each sex within the group. The heatmap on the right column was graphically represented using the modified t-values calculated from the two-sample *t*-test, assuming unequal variances for differences in fractional anisotropy values between male and female rats. Significant fractional anisotropy differences in structural connectivities are marked with single or double asterisks; *P < 0.05, **P < 0.01 (*n*  *=* 27 males; *n*  *=* 30 females, ACC, anterior cingulate cortex; Amy, amygdala; IC, insular cortex; NAcc, nucleus accumbens; PAG, periaqueductal grey; PFC, prefrontal cortex; S1, primary somatosensory cortex; S2, secondary somatosensory cortex; VP, ventral posterior thalamic nucleus (detailed data in Supplementary Table 3).

DTI data were obtained from the sham, NP and ICS model groups and were subjected to analysis. Representative images of each group are shown in Fig. 2. To analyse the data, we evaluated the connectivity of nine brain regions [ventral posterior thalamic nucleus (VP), ACC, amygdala (Amy), IC, nucleus accumbens (NAcc), PAG, PFC, primary somatosensory cortex (S1) and secondary somatosensory cortex (S2)] by sex in the Sham, NP and ICS groups. Initially, we assessed the degree of structural connectivity between the brain regions in each group using FA values. In the Sham group, females exhibited significantly higher structural connectivity in seven interregional measures than males. The tracts showing significant differences typically included the ACC–NAcc, Amy–NAcc, Amy-PAG and Amy-PFC. Additionally, significant differences in FA values were observed in the connections between the NAcc and VP, PAG-VP and PFC-VP (Figure 3A). These results indicate that the structural connectivity in the limbic system, especially the amygdala, to other reasons is higher in females than in males. In the NP group, only the tract between Amy-VP showed differences in FA values between males and females (Fig. 3B). Finally, in the ICS group, six connections showing higher FA values in males than in females were observed between the ACC-VP, IC-S1, IC-VP, NAcc-VP, PFC-S1 and S1-VP (Fig. 3C and Supplementary Tables 1 and 2). To visualize the FA values in the pathways between the two regions and identify potential changes, we employed a heatmap visualization method (Figure 4 and Supplementary Table 4). Darker colors represent higher FA values, and lighter colors correspond to lower FA values. In addition, to compare the qualitative differences between males and females, we employed an analytical approach that examined the value differences represented in the heatmap. In the analysis of the FA differences between males and females, the connection between ACC-NAcc showed the greatest difference and the connection between the Amy-NAcc, Amy-PAG and Amy-PFC, and the connection between NAcc-VP, PAG-VP and PFC-VP showed that females had higher FA values (Fig. 4A). Although lower FA value differences were observed in both males and females within the NP group, the connection between Amy-VP was higher in females (Fig. 4B). In the ICS group, the change in FA in the brain was higher in males than in females, and the greatest difference was observed in the IC-S1, IC-VP and NAcc-VP pathways. The connection between the PFC-S1 and S1-VP pathways showed significantly higher FA values (Fig. 4C). These findings suggest that there are differences in basic brain connectivity between males and females in brain regions related to pain perception and that there are differences in brain microstructural organization and connectivity after chronic pain and pain relief. Detailed FA values are provided in Supplementary Table 3.

**Figure 5:**
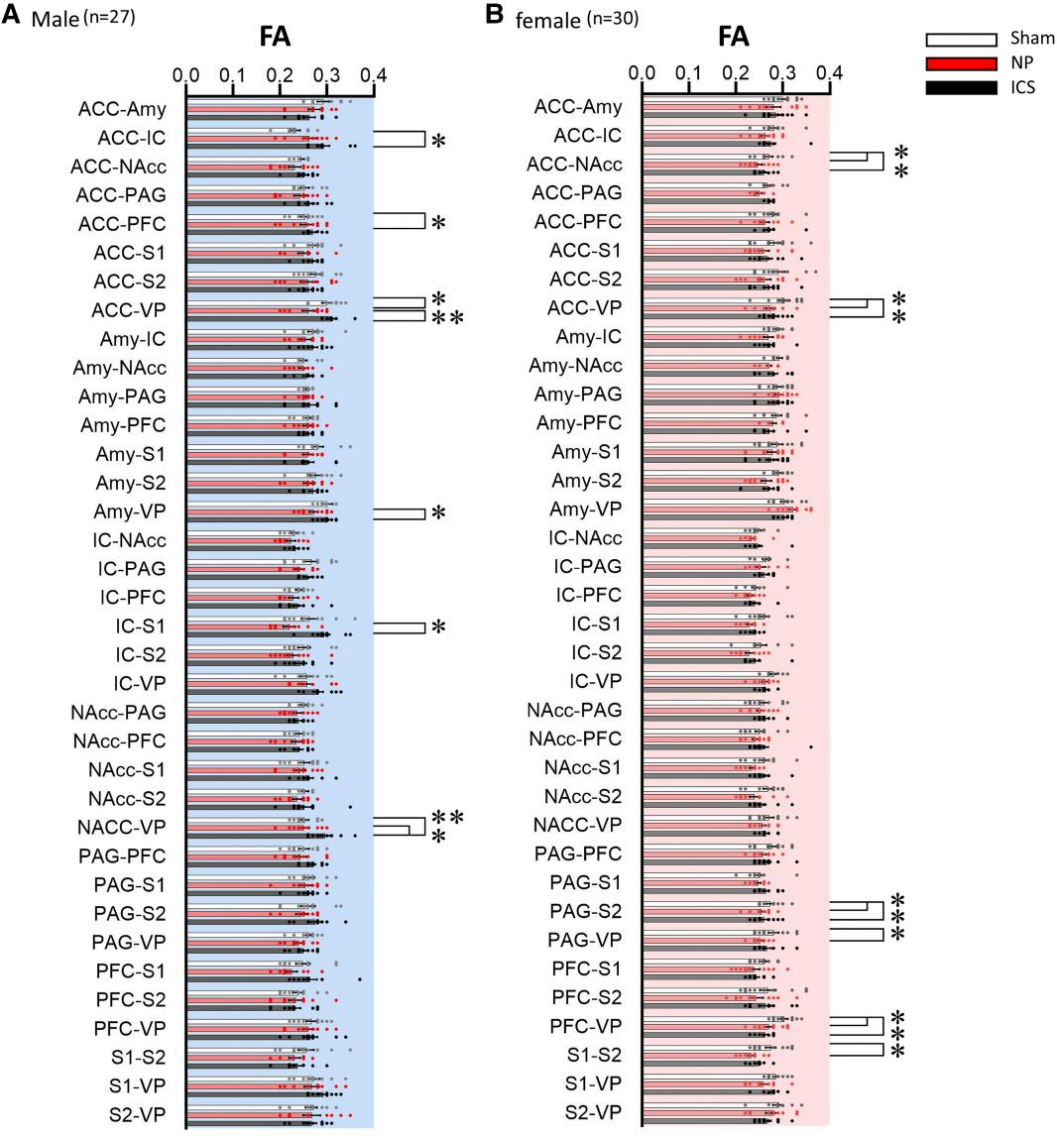
**Sex-based fractional anisotropy comparison among Sham, NP and insular cortex stimulation.** Comparison of fractional anisotropy values in functional connectivity among Sham, NP and insular cortex stimulation groups in male **(A)** and female **(B)** rats. The white, red, and black bars represent the mean fractional anisotropy values for the Sham, NP and insular cortex stimulation groups, respectively. Each data point represents an individual animal. Error bars correspond to the SEM. Statistical significance was assessed by 1-way ANOVA and Tukey’s HSD *post hoc* test: *P < 0.05, **P < 0.01 (*n*  *=* 27 males; *n*  *=* 30 females). ACC, anterior cingulate cortex; Amy, amygdala; IC, insular cortex; NAcc, nucleus accumbens; PAG, periaqueductal grey; PFC, prefrontal cortex; S1, primary somatosensory cortex; S2, secondary somatosensory cortex; VP, ventral posterior thalamic nucleus (detailed data in Supplementary Table 4).

To determine whether the differences in FA values between the groups reflected variations in brain connectivity, we compared the FA changes between males and females. In the male group, the only connection showing a significant FA difference between the Sham and NP groups was the pathway between the ACC-VP, which exhibited a decrease in FA in the NP group. In the ICS group, connections showing increased FA values compared to the NP group included the pathways between the ACC-VP, Amy-VP, IC-S1 and NAcc-VP. Additionally, connections with significantly higher FA values in the ICS group than in the sham group were observed in the ACC-IC, ACC-PFC and NAcc-VP (Fig. 5A and Supplementary Table 4). In females, connectivity generally showed lower FA values in both the NP and ICS groups than in the Sham group. Specifically, significant reductions in FA values were observed in the NP group compared to the Sham group in the connections between the ACC-NAcc, ACC-VP, PAG-S2, PAG-VP, PFC-VP and S1-S2. Furthermore, even after pain was alleviated following ICS, the connections between the ACC-NAcc, ACC-VP, PAG-S2 and PFC-VP continued to exhibit reduced FA values compared to the Sham group (Fig. 5B, Supplementary Table 4).

**Figure 6:**
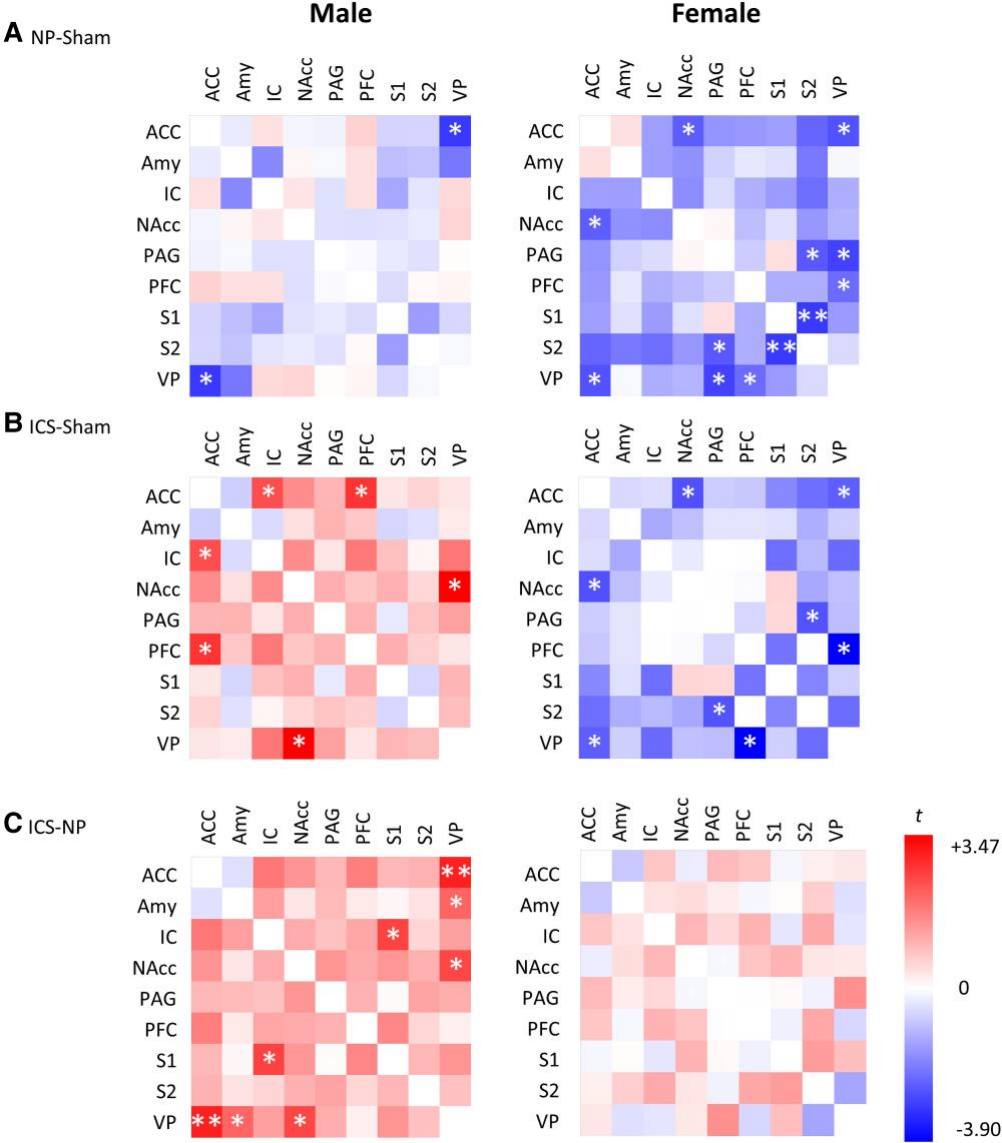
**Pairwise multiple comparisons of fractional anisotropy values in functional connectivity among Sham, NP and insular cortex stimulation groups in each sex**. (**A–C**) The differences between the fractional anisotropy values in the insular cortex stimulation insular cortex stimulation and NP groups. The fractional anisotropy differences in the insular cortex stimulation-NP between males and females were analysed. The intensity of the red color indicates a higher change in association between insular cortex stimulation–related brain regions after insular cortex stimulation, whereas a darker blue color indicates a higher correlation between pain-related brain regions after insular cortex stimulation. Significant fractional anisotropy differences in structural connectivities are marked with single or double asterisks; *P < 0.05, **P < 0.01 (*n*  *=* 27 males; *n*  *=* 30 females). ACC, anterior cingulate cortex; Amy, amygdala; IC, insular cortex; NAcc, nucleus accumbens; PAG, periaqueductal grey; PFC, prefrontal cortex; S1, primary somatosensory cortex; S2, secondary somatosensory cortex; VP, ventral posterior thalamic nucleus. Detailed data in Supplementary Table 5.

To evaluate the degree of change in brain connectivity within each group, FA values were compared across groups to calculate the magnitude of change. These changes were visualized as a heatmap to illustrate the relative differences (Fig. 6 and Supplementary Table 5). To identify the reductions in brain connectivity associated with the pain state, we employed an analytical method that involved subtracting the FA values of the NP group from those of the Sham group. In the analysis results, male rats showed a significant difference only in the connection between ACC-VP. In contrast, female rats showed significant differences in the connections between ACC-NAcc, ACC-VP, PAG-S2, PAG-VP, PFC-VP and S1-S2 (Fig. 6A). To assess the extent to which brain region connectivity deviates from the normal state following ICS, we analysed the changes using the differences in FA values between the Sham and ICS groups. In male rats, the connections between the ACC-IC, ACC-PFC and NAcc-VP were significantly stronger in the ICS than in the Sham group. However, in female rats, the FA value of the ICS was lower than that of Sham, so it showed an overall negative value, and there was a significant difference in the connection between the ACC-NAcc, Acc-VP, PAG-S2 and PFC-VP (Fig. 6B). Finally, we analysed the differences in FA values between the ICS and NP groups to determine whether ICS increased the FA values between brain regions in the pain state. Significant differences were observed exclusively in the ACC-VP, Amy-VP, IC-S1 and NAcc-VP pathways in male rats. In contrast, no significant differences in FA values were detected between the ICS and NP groups in female rats (Figure 6C). The detailed subtracted FA data are presented in Supplementary Table 5.

### Statistical analysis of the relationship between pain severity and brain structural changes

**Figure 7:**
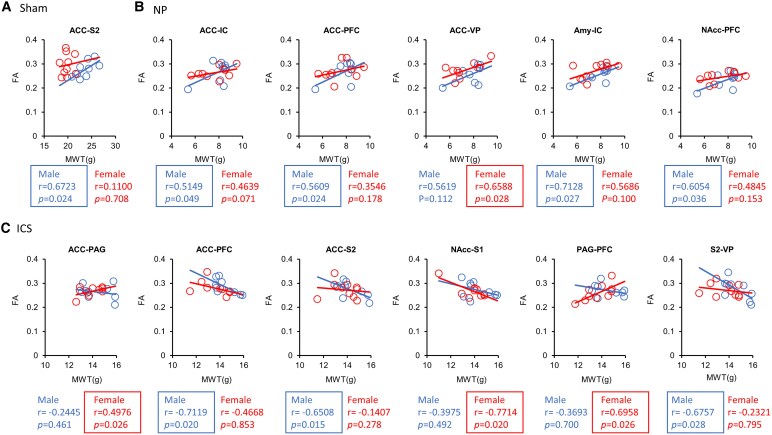
**Scatter plot analysis of fractional anisotropy–mechanical withdrawal threshold correlation.** Scatter plot demonstrating the significant correlation between fractional anisotropy values in functional connectivity and mechanical withdrawal threshold (g) in the Sham (**A**), NP (**B**) and insular cortex stimulation (**C**) groups. The circles, triangles and squares in the plots represent the Sham, NP and insular cortex stimulation groups, respectively. Each data point represents an individual animal and blue and red colors represent data from male and female rats, respectively. Pearson’s correlation coefficients (*r*), degrees of freedom (*n*-2) and *P-*values are shown at the bottom of the *x*-axis of each plot. *P < 0.05 indicates a significant correlation (*n*  *=* 27 males; *n*  *=* 30 females, detailed data in Supplementary Table 6).

Finally, we examined the relationship between the FA values across brain regions and the observed pain levels, as determined by behavioural tests, using a linear correlation model. Spearman correlation coefficients were calculated to assess the relationship between the measured MW and FA values for each of the Sham, NP and ICS groups (Fig. 7 and Supplementary Table 6). In the Sham group, a significant correlation between the MWT and FA values in the tracts connecting the ACC-S2 was found only in male rats (ACC and S2: *r* = 0.6723, *P* = 0.024). In the NP group, a significant positive correlation between the MWT and FA values was found in the tracts between the ACC-IC, ACC-PFC, Amy-IC and NAcc-PFC (ACC-IC: *r* = 0.5149, *P* = 0.049; ACC-PFC: *r* = 0.5609, *P* = 0.024, Amy-IC: *r* = 0.7128, *P* = 0.027 and NAcc-PFC: *r* = 0.6054, *P* = 0.036). In female rats, there were significant correlations between the MWT and FA values in the ACC-VP tract (ACC-VP: *r* = 0.6588, *P* = 0.028). In the ICS group, male rats showed significant differences in the ACC-PFC, ACC-S2, and S2-VP (ACC-PFC: *r* = −0.7119, *P* = 0.020; ACC-S2: *r* = −0.6508, *P* = 0.015, and S2-VP: *r* = −0.6757, *P* = 0.028). Female rats exhibited significant correlations between the MWT and FA values in the ACC-PAG, NAcc-S1 and PAG-PFC tracts (ACC-PAG: *r* = 0.4976, *P* = 0.026; NAcc-S1: *r* = −0.7714, *P* = 0.020 and PAG-PFC: *r* = 0.6958, *P* = 0.026). These findings suggest that pain is associated with enhanced connectivity between different brain regions. Furthermore, the observed changes in connectivity following ICS treatment revealed distinct sex differences in brain network modulation.

## Discussion

The present study explored sex-specific differences in brain activation and pain modulation pathways during chronic pain management after ICS use. Structural connectivity was analysed using a pain model and DTI. In chronic pain conditions, males exhibited decreased FA values between the ACC-VP, whereas females showed reduced FA values across multiple pathways, including the ACC–NAcc, ACC–VP, PAG–S2, PAG–VP, PFC–VP and S1–S2. Pain relief in males was associated with increased FA values in the ACC–VP, Amy–VP, IC–S1 and NAcc–VP pathways. In contrast, females showed no significant FA differences between the NP and ICS groups. These findings highlight clear sex-related differences in neural activation and pain modulation mechanisms, emphasizing the need for sex-specific approaches in chronic pain research and treatment development. This study provides new insights into the changes in structural connectivity underlying pain and its modulation, offering a foundation for personalized neuromodulation strategies.

Chronic pain typically affects >20% of adults and is a major contributor to impairment worldwide.^44^ In addition, even when the type of pain is the same, the degree of pain perception often differs between males and females.^45^ The existence of sex differences in the prevalence of chronic pain has been reported in many studies.^46-50^ Females are reported to have a higher likelihood of experiencing other conditions associated with chronic pain and to exhibit greater sensitivity to pain than males.^47^ Our previous studies have demonstrated sex differences in pain perception using behavioural assessments,^45^ and our present results provide further evidence that ICS effectively restores pain thresholds in both males and females and that the mechanisms of this restoration are mediated by different brain activations. Human studies using fMRI and PET have also revealed sex differences in brain structure formation, neural activation and connectivity.^5,50,51^ The IC is a structurally and functionally heterogeneous region, with its subregions contributing to pain perception and modulation. While the posterior IC has been primarily implicated in encoding the sensory-discriminative aspects of nociceptive stimuli, stimulation of the anterior IC has been associated with the affective, cognitive, and anticipatory dimensions of pain.^52,53^ Notably, the anterior IC forms direct reciprocal connections with key regions involved in descending pain control, including the ACC, mPFC, Amy and PAG.^54^ These anatomical projections support the role of the anterior IC as a hub that integrates interoceptive awareness with the top-down modulation of nociceptive input. Therefore, in the present study, we specifically targeted the rostral anterior IC to engage this broader pain regulatory network, rather than the primary sensory nociceptive pathways.

In this study, we demonstrated that the connectivity between the Amy and PFC was significantly higher in female rats than in male rats in the sham group, as evidenced by quantitative increases in FA values measured using DTI (Fig. 3A). Consistent with primate studies that have reported the involvement of the amygdala and PFC in emotional processing and cognitive functions such as pain-related memory and stimulus evaluation,^55,56^ our findings revealed sex-dependent differences in the connectivity of these regions. While previous studies have explored sex differences in pain sensitivity by examining biological mechanisms, including hormonal regulation and immunological factors, alongside psychosocial influences such as social support and emotion-focused interventions,^57-59^ these mechanisms remain incompletely understood and poorly characterized. Further research is warranted to investigate sex-specific neural responses to nociceptive stimuli and the functional connectivity between brain regions, which may provide deeper insights into the mechanisms underlying sex-specific pain processing.^60,61^ Notably, our results indicate that these sex-dependent differences in brain connectivity became negligible in most cases during the pain state, except for the FA values of the Amy-VP pathway. Interestingly, in response to ICS, FA values were higher in male rats than in females, reflecting significant sex differences in the connectivity between regions directly and indirectly associated with ICS. An interesting finding from our study was the enhanced activation of the pathway connecting the ACC, IC, PFC, S1 and VP in males compared to that in females following ICS (Figs 4C and 6C). This pronounced sex difference in pain modulation suggests that ICS enhances these pathways more strongly in males than in females. This effect was corroborated by more distinct FA changes and intergroup differences observed in the heatmap analyses. Previous studies on sex differences in pain modulation via the ICS have highlighted significant sex-specific characteristics in pain processing within the IC.^62^ These differences are attributed to the distinct structural and functional characteristics of pyramidal neurons in the IC; however, further research is needed to explore these sex-specific features in greater detail.^62,63^ When analysing the FA changes across the three groups (Sham, NP and ICS) in both males and females, the results revealed distinct patterns were observed. In males, FA values increased following ICS in pathways such as the AA-IC, ACC-PFC and NAcc-VP, with significant intergroup differences observed in the ACC-VP, Amy-VP, IC-S1 and NAcc-VP. Conversely, in females, FA values tended to decrease following ICS in pathways, including the ACC-NAcc, ACC-VP, PAG-S2 and PFC-VP. Notably, connections in the NP group exhibited lower FA values than those in the Sham group, particularly in the ACC-NAcc, AVV-VP, PAG-S2 and PFC-VP. These findings indicate that different incertothalamic pathways may be involved in gender-specific pain modulation processes following ICS.^64-66^ Albanese *et al*.^67^ and Bornhovd *et al*.^68^ demonstrated that the anterior IC, ACC and medial prefrontal cortex are strongly associated with the subjective experience of pain, whereas the somatosensory cortex is more closely linked to the intensity of the stimulus. Our findings corroborate these results. Consistent with prior experimental studies, the IC, a key component of the limbic system, has been implicated in the emotional processing of pain, as evidenced by activation observed in PET and fMRI studies of various pain types.^69-71^ In our study, we did not perform whole-brain DTI analysis because our primary objective was to investigate distinct changes in the brain regions contralateral to the nerve injury, where pain signals are primarily processed. The observed changes in structural connectivity levels, reflected by fluctuations in FA values, along with the increase in pain threshold following direct stimulation of the IC, are significant and warrant further investigation.

DTI is a noninvasive MRI technique that quantifies both isotropic and anisotropic water diffusion in tissues, offering valuable insights into the microstructural integrity and connectivity within the brain.^23,72,73^ This technique has been increasingly applied to identify potential biomarkers of chronic pain and assess treatment outcomes. In this study, we investigated sex-specific differences in the degree of connectivity across nine brain regions that are implicated in pain processing. Notably, differences in FA values reflected substantial changes in the strength of pain-related connectivity, with enhanced brain connectivity following ICS demonstrating significant sex differences (Figs 4 and 5). These findings provide compelling evidence that the limbic system plays a critical role in pain regulation, exhibiting distinct patterns of modulation between males and females. The relationship between the limbic system and pain processing has been extensively studied.^74,75^ Within this system, Amy and PAG are central to the modulation of fear and anxiety. The Amy not only processes sensory stimuli, including pain but also transmits this information to surrounding areas, enabling the organism to adapt to specific environmental conditions.^56,74^ Upon receiving inputs from the Amy, the PAG initiates physical responses to manage fear and stress, whereas other brain regions modulate these responses by regulating PAG activity.^76^

Another critical component of the limbic system, the NAcc, plays a key role in regulating emotions, such as pleasure, reward, and motivation, while also influencing behaviour.^77^ In addition to these functions, the NAcc is intricately involved in pain processing and modulation.^78,79^ The connections between the core and shell of the NAcc form a feedback loop essential for pain processing, suggesting that the NAcc integrates nociceptive inputs and disseminates signals throughout the pain processing network.^78-80^ Given that pain serves as an aversive stimulus to promote avoidance and adaptive behaviours, its encoding and memory rely on neural circuits associated with motivation and negative reinforcement learning.^81^ The integration of the sensory and emotional components of pain likely involves strengthened connectivity between key regions.^82^ For instance, the secondary S2, which integrates sensory information for decision-making, may exhibit enhanced connectivity with the Amy and PFC, which are regions central to pain processing and negative reinforcement. These strengthened connections may facilitate adaptive responses to painful stimuli, underscoring the intricate interplay between the sensory, emotional, and cognitive networks in pain modulation.

The results of the behavioural tests and correlation analysis of FA provide strong support for our hypothesis. While robust sensory-emotional connectivity between the ACC and S2 was observed in sham males, in the NP condition, connectivity between the ACC, IC, PFC, Amy and NAcc was strongly correlated with pain in male rats. In contrast, in females, only ACC-VP connectivity exhibited changes associated with the pain behaviour. These brain regions are critical for processing the sensory, emotional, and cognitive dimensions of pain, and their interactions contribute to the regulation of emotional responses to pain.^83,84^ These findings suggest alterations in both the pain processing network and the emotional-motivational processing network related to pain behaviours. Additionally, changes in the IC showed negative correlations in males between the ACC-PFC, ACC-S2 and S2-VP, whereas in females, negative correlations were observed in the NAcc-S1. Conversely, positive correlations were found between the ACC-PAG and PAG-PFC in females. These results indicate modifications within the pain processing network, affective-motivational processing network, or sensory-affective network, highlighting the complex integration of sensory, affective, and cognitive processing in generating a comprehensive pain response. Specifically, the ACC and IC process the affective and sensory components of pain,^85,86^ the Amy and NAcc enhance emotional and motivational responses,^87,88^ and the PFC modulates these responses cognitively to induce appropriate behavioural responses.^89^

The present findings demonstrate that ICS elicits measurable changes in both pain-related behaviours and white matter connectivity, as assessed using FA-based tractography. These results have potential translational significance, particularly given the conserved role of the rostral IC in nociceptive processing across species. In humans, the insula is critically involved in the affective-motivational and interoceptive dimensions of pain, and its altered activity and connectivity have been robustly reported in various chronic pain conditions, including neuropathic pain, fibromyalgia, and complex regional pain syndrome (CRPS). Thus, the observed effects of ICS in this preclinical model may inform the development of targeted neuromodulatory strategies aimed at the IC or IC-associated circuits for clinical pain management. In addition, the alterations in FA-based connectivity observed following ICS suggest the engagement of broader circuit-level mechanisms potentially underlying its analgesic effects. These may include (i) descending pain modulatory pathways, particularly involving projections from the IC to the PAG; (ii) limbic-cortical integration, encompassing altered connectivity among the IC, ACC, PFC, and NAcc, which are implicated in the emotional-cognitive regulation of pain; and (iii) thalamocortical plasticity, which may reflect reorganization of somatosensory input pathways following chronic injury. Collectively, these circuit-level changes may represent a form of adaptive plasticity contributing to pain relief, thereby providing a mechanistic basis for observed behavioural outcomes. These insights highlight the relevance of insula-targeted neuromodulation not only in experimental paradigms but also in translational contexts, offering a promising direction for non-opioid, circuit-specific interventions in chronic pain therapy.

Although FA is known to exhibit low inter-subject variability and high test–retest reliability,^90,91^ we considered the potential confounding effects of individual baseline differences when interpreting group-level differences in FA values across the Sham, NP and ICS conditions. Although random group assignment and the absence of significant pre-intervention differences in DTI metrics reduce the likelihood of systematic bias, it is still possible that pre-existing differences in white matter microstructures contributed to the observed post-intervention FA changes. Ideally, normalizing FA values to each subject’s baseline would yield more accurate estimates of treatment effects. Although fully longitudinal DTI acquisition was not feasible for all animals in the present study, this limitation underscores the importance of incorporating pre- and post-intervention imaging in future studies to control individual variability more effectively.

Our study primarily focused on changes in brain connectivity in rats, specifically investigating sex differences in connectivity following pain modulation using the ICS. It is crucial to recognize that sex differences influence brain connectivity in the perception and regulation of pain. These findings offer compelling evidence for the need to consider sex as a factor in clinical pain management, potentially guiding the development of sex-specific therapeutic strategies in the future. Through our detailed DTI analysis, we provide critical insights into the alterations in connectivity across various brain regions during pain processing and its modulation. We anticipate that this study will contribute significantly to the understanding of sex-based differences in pain perception and serve as a foundation for future studies exploring the neural mechanisms underlying effective pain treatment.

## Supplementary Material

fcaf362_Supplementary_Data

## Data Availability

The data underlying this article are available in the article and in its online supplementary material. The custom code used for data analysis in this study has been uploaded to an online repository and is available at the following link: (https://gist.github.com/myeounghooncha/361d611843aa1a6522869e838be33a47.js).

## References

[1] Fillingim RB, Maixner W. Gender differences in the responses to noxious stimuli. Pain Forum. 1995;4(4):209–221.

[2] Robinson ME, Wise EA, Riley JL, Atchison JW. Sex differences in clinical pain: A multisample study. J Clin Psychol Med Settings. 1998;5(4):413–424.

[3] Umeda M, Okifuji A. Exploring the sex differences in conditioned pain modulation and its biobehavioral determinants in healthy adults. Musculoskelet Sci Pract. 2023;63:102710. 36566112 10.1016/j.msksp.2022.102710

[4] Beery AK, Zucker I. Sex bias in neuroscience and biomedical research. Neurosci Biobehav Rev. 2011;35(3):565–572. 20620164 10.1016/j.neubiorev.2010.07.002 PMC3008499

[5] Hagiwara H, Sakimura K, Abe M, et al Sex differences in pain-induced modulation of corticotropin-releasing hormone neurons in the dorsolateral part of the stria terminalis in mice. Brain Res. 2021;1773:147688. 34644526 10.1016/j.brainres.2021.147688

[6] Bouhassira D, Lantéri-Minet M, Attal N, Laurent B, Touboul C. Prevalence of chronic pain with neuropathic characteristics in the general population. Pain. 2008;136(3):380–387. 17888574 10.1016/j.pain.2007.08.013

[7] Mapplebeck JCS, Beggs S, Salter MW. Sex differences in pain: A tale of two immune cells. Pain. 2016;157(Suppl 1):S2–s6. 26785152 10.1097/j.pain.0000000000000389

[8] Sorge RE, Strath LJ. Sex differences in pain responses. Curr Opin Physiol. 2018;6:75–81.

[9] Sorge RE, Totsch SK. Sex differences in pain. J Neurosci Res. 2017;95(6):1271–1281. 27452349 10.1002/jnr.23841

[10] Menzler K, Belke M, Wehrmann E, et al Men and women are different: Diffusion tensor imaging reveals sexual dimorphism in the microstructure of the thalamus, corpus callosum and cingulum. Neuroimage. 2011;54(4):2557–2562. 21087671 10.1016/j.neuroimage.2010.11.029

[11] Koolschijn PC, Crone EA. Sex differences and structural brain maturation from childhood to early adulthood. Dev Cogn Neurosci. 2013;5:106–118. 23500670 10.1016/j.dcn.2013.02.003 PMC6987760

[12] Cook KM, De Asis-Cruz J, Lopez C, et al Robust sex differences in functional brain connectivity are present in utero. Cereb Cortex. 2023;33(6):2441–2454. 35641152 10.1093/cercor/bhac218 PMC10016060

[13] Yang X, Li A, Li L, Li T, Li P, Liu M. Multimodal image analysis of sexual dimorphism in developing childhood brain. Brain Topogr. 2021;34(3):257–268. 33630209 10.1007/s10548-021-00823-7

[14] van Eijk L, Zhu D, Couvy-Duchesne B, et al Are sex differences in human brain structure associated with sex differences in behavior? Psychol Sci. 2021;32(8):1183–1197. 34323639 10.1177/0956797621996664 PMC8726594

[15] Ko JH, Tang CC, Eidelberg D. Brain stimulation and functional imaging with fMRI and PET. Handb Clin Neurol. 2013;116:77–95. 24112887 10.1016/B978-0-444-53497-2.00008-5

[16] Cosgrove KP, Mazure CM, Staley JK. Evolving knowledge of sex differences in brain structure, function, and chemistry. Biol Psychiatry. 2007;62(8):847–855. 17544382 10.1016/j.biopsych.2007.03.001 PMC2711771

[17] Kawano H, Yamada S, Watanabe Y, et al Aging and sex differences in brain volume and cerebral blood flow. Aging Dis. 2023;15(5):2216–2229. 10.14336/AD.2023.1122 PMC11346398 38029394

[18] Kawachi T, Ishii K, Sakamoto S, Matsui M, Mori T, Sasaki M. Gender differences in cerebral glucose metabolism: A PET study. J Neurol Sci. 2002;199(1–2):79–83. 12084447 10.1016/s0022-510x(02)00112-0

[19] Armstrong NM, Huang CW, Williams OA, et al Sex differences in the association between amyloid and longitudinal brain volume change in cognitively normal older adults. Neuroimage Clin. 2019;22:101769. 30927602 10.1016/j.nicl.2019.101769 PMC6444285

[20] Zhang X, Liang M, Qin W, Wan B, Yu C, Ming D. Gender differences are encoded differently in the structure and function of the human brain revealed by multimodal MRI. Front Hum Neurosci. 2020;14:244. 32792927 10.3389/fnhum.2020.00244 PMC7385398

[21] Ebel M, Domin M, Neumann N, Schmidt CO, Lotze M, Stanke M. Classifying sex with volume-matched brain MRI. Neuroimage Rep. 2023;3(3):100181. 40567385 10.1016/j.ynirp.2023.100181 PMC12172721

[22] Cavaliere C, Aiello M, Di Perri C, Fernandez-Espejo D, Owen AM, Soddu A. Diffusion tensor imaging and white matter abnormalities in patients with disorders of consciousness. Front Hum Neurosci. 2015;8:1028. 25610388 10.3389/fnhum.2014.01028 PMC4285098

[23] Huisman TA, Schwamm LH, Schaefer PW, et al Diffusion tensor imaging as potential biomarker of white matter injury in diffuse axonal injury. AJNR Am J Neuroradiol. 2004;25(3):370–376. 15037457 PMC8158566

[24] Oni MB, Wilde EA, Bigler ED, et al Diffusion tensor imaging analysis of frontal lobes in pediatric traumatic brain injury. J Child Neurol. 2010;25(8):976–984. 20332386 10.1177/0883073809356034 PMC3227397

[25] Bao H, Li R, He M, Kang D, Zhao L. DTI study on brain structure and cognitive function in patients with chronic mountain sickness. Sci Rep. 2019;9(1):19334. 31852992 10.1038/s41598-019-55498-9 PMC6920146

[26] Dimov LF, Toniolo EF, Alonso-Matielo H, et al Electrical stimulation of the insular cortex as a novel target for the relief of refractory pain: An experimental approach in rodents. Behav Brain Res. 2018;346:86–95. 29191577 10.1016/j.bbr.2017.11.036

[27] Craig AD . How do you feel? Interoception: The sense of the physiological condition of the body. Nat Rev Neurosci. 2002;3(8):655–666. 12154366 10.1038/nrn894

[28] Mazzola L, Isnard J, Peyron R, Guénot M, Mauguière F. Somatotopic organization of pain responses to direct electrical stimulation of the human insular cortex. PAIN. 2009;146(1):99–104. 19665303 10.1016/j.pain.2009.07.014

[29] Tracey I, Mantyh PW. The cerebral signature for pain perception and its modulation. Neuron. 2007;55(3):377–391. 17678852 10.1016/j.neuron.2007.07.012

[30] Kim K, Nan G, Kim L, et al Insular cortex stimulation alleviates neuropathic pain via ERK phosphorylation in neurons. CNS Neurosci Ther. 2023;29(6):1636–1648. 36806498 10.1111/cns.14126 PMC10173725

[31] Kobayashi S, O'Hashi K, Kobayashi M. Repetitive nociceptive stimulation increases spontaneous neural activation similar to nociception-induced activity in mouse insular cortex. Sci Rep. 2022;12(1):15190. 36071208 10.1038/s41598-022-19562-1 PMC9452502

[32] Gogolla N . The insular cortex. Curr Biol. 2017;27(12):R580–R5r6. 28633023 10.1016/j.cub.2017.05.010

[33] Kim K, Nan G, Kim HY, Cha M, Lee BH. Targeting the insular cortex for neuropathic pain modulation: Insights into synaptic and neuronal mechanisms. Faseb J. 2025;39(2):e70285. 39831885 10.1096/fj.202402381R PMC11745213

[34] Kim K, Nan G, Bak H, et al Insular cortex stimulation alleviates neuropathic pain through changes in the expression of collapsin response mediator protein 2 involved in synaptic plasticity. Neurobiol Dis. 2024;194:106466. 38471625 10.1016/j.nbd.2024.106466

[35] Coffeen U, Manuel Ortega-Legaspi J, Lopez-Munoz FJ, Simon-Arceo K, Jaimes O, Pellicer F. Insular cortex lesion diminishes neuropathic and inflammatory pain-like behaviours. Eur J Pain. 2011;15(2):132–138. 20619707 10.1016/j.ejpain.2010.06.007

[36] Garcia R, Simon MJ, Puerto A. Conditioned place preference induced by electrical stimulation of the insular cortex: Effects of naloxone. Exp Brain Res. 2013;226(2):165–174. 23377149 10.1007/s00221-013-3422-7

[37] Lee BH, Won R, Baik EJ, Lee SH, Moon CH. An animal model of neuropathic pain employing injury to the sciatic nerve branches. Neuroreport. 2000;11(4):657–661. 10757496 10.1097/00001756-200003200-00002

[38] Schilling KG, Yeh FC, Nath V, et al A fiber coherence index for quality control of B-table orientation in diffusion MRI scans. Magn Reson Imaging. 2019;58:82–89. 30682379 10.1016/j.mri.2019.01.018 PMC6401245

[39] Barriere DA, Magalhaes R, Novais A, et al The SIGMA rat brain templates and atlases for multimodal MRI data analysis and visualization. Nat Commun. 2019;10(1):5699. 31836716 10.1038/s41467-019-13575-7 PMC6911097

[40] Paxinos G, Watson C. The rat brain in stereotaxic coordinates. 6th ed. Academic Press; 2005. 10.1016/0165-0270(80)90021-7 6110810

[41] Tournier JD, Smith R, Raffelt D, et al MRtrix3: A fast, flexible and open software framework for medical image processing and visualisation. Neuroimage. 2019;202:116137. 31473352 10.1016/j.neuroimage.2019.116137

[42] Zhang Y, Vakhtin AA, Jennings JS, et al Diffusion tensor tractography of brainstem fibers and its application in pain. PLoS One. 2020;15(2):e0213952. 32069284 10.1371/journal.pone.0213952 PMC7028272

[43] Raji CA, Wang MB, Nguyen N, et al Connectome mapping with edge density imaging differentiates pediatric mild traumatic brain injury from typically developing controls: Proof of concept. Pediatr Radiol. 2020;50(11):1594–1601. 32607611 10.1007/s00247-020-04743-9 PMC7501221

[44] Goldberg DS, McGee SJ. Pain as a global public health priority. BMC Public Health. 2011;11:770. 21978149 10.1186/1471-2458-11-770 PMC3201926

[45] Cha M, Eum YJ, Kim K, et al Diffusion tensor imaging reveals sex differences in pain sensitivity of rats. Front Mol Neurosci. 2023;16:1073963. 36937048 10.3389/fnmol.2023.1073963 PMC10017469

[46] Zhou JZ, Deng J, Luo DX, et al Sex differences in functional and structural alterations of hippocampus region in chronic pain: A DTI and resting-state fMRI study. Front Neurosci. 2024;18:1428666. 39308951 10.3389/fnins.2024.1428666 PMC11412943

[47] Mogil JS . Qualitative sex differences in pain processing: Emerging evidence of a biased literature. Nat Rev Neurosci. 2020;21(7):353–365. 32440016 10.1038/s41583-020-0310-6

[48] Cabañero D, Villalba-Riquelme E, Fernández-Ballester G, Fernández-Carvajal A, Ferrer-Montiel A. ThermoTRP channels in pain sexual dimorphism: New insights for drug intervention. Pharmacol Ther. 2022;240:108297. 36202261 10.1016/j.pharmthera.2022.108297

[49] Nasser SA, Afify EA. Sex differences in pain and opioid mediated antinociception: Modulatory role of gonadal hormones. Life Sci. 2019;237:116926. 31614148 10.1016/j.lfs.2019.116926

[50] Mogil JS . Sex differences in pain and pain inhibition: Multiple explanations of a controversial phenomenon. Nat Rev Neurosci. 2012;13(12):859–866. 23165262 10.1038/nrn3360

[51] Monroe TB, Fillingim RB, Bruehl SP, et al Sex differences in brain regions modulating pain among older adults: A cross-sectional resting state functional connectivity study. Pain Med. 2018;19(9):1737–1747. 28505337 10.1093/pm/pnx084 PMC6454788

[52] Zhang R, Deng H, Xiao X. The insular Cortex: An interface between sensation, emotion and cognition. Neurosci Bull. 2024;40(11):1763–1773. 38722464 10.1007/s12264-024-01211-4 PMC11607240

[53] Mercer Lindsay N, Chen C, Gilam G, Mackey S, Scherrer G. Brain circuits for pain and its treatment. Sci Transl Med. 2021;13(619):eabj7360. 34757810 10.1126/scitranslmed.abj7360 PMC8675872

[54] Kayyal H, Chandran SK, Yiannakas A, Gould N, Khamaisy M, Rosenblum K. Insula to mPFC reciprocal connectivity differentially underlies novel taste neophobic response and learning in mice. Elife. 2021;10:e66686. 34219650 10.7554/eLife.66686 PMC8282338

[55] Strigo IA, Duncan GH, Boivin M, Bushnell MC. Differentiation of visceral and cutaneous pain in the human brain. J Neurophysiol. 2003;89(6):3294–3303. 12611986 10.1152/jn.01048.2002

[56] Gandhi W, Rosenek NR, Harrison R, Salomons TV. Functional connectivity of the amygdala is linked to individual differences in emotional pain facilitation. Pain. 2020;161(2):300–307. 31613866 10.1097/j.pain.0000000000001714

[57] Cairns BE, Gazerani P. Sex-related differences in pain. Maturitas. 2009;63(4):292–296. 19595525 10.1016/j.maturitas.2009.06.004

[58] Craft RM . Modulation of pain by estrogens. Pain. 2007;132(Suppl 1):S3–s12. 17951003 10.1016/j.pain.2007.09.028

[59] Racine M, Tousignant-Laflamme Y, Kloda LA, Dion D, Dupuis G, Choinière M. A systematic literature review of 10 years of research on sex/gender and pain perception—Part 2: Do biopsychosocial factors alter pain sensitivity differently in women and men? Pain. 2012;153(3):619–635. 22236999 10.1016/j.pain.2011.11.026

[60] Derbyshire SW, Nichols TE, Firestone L, Townsend DW, Jones AK. Gender differences in patterns of cerebral activation during equal experience of painful laser stimulation. J Pain. 2002;3(5):401–411. 14622744 10.1054/jpai.2002.126788

[61] Straube T, Schmidt S, Weiss T, Mentzel HJ, Miltner WH. Sex differences in brain activation to anticipated and experienced pain in the medial prefrontal cortex. Hum Brain Mapp. 2009;30(2):689–698. 18219622 10.1002/hbm.20536 PMC6870921

[62] Iezzi D, Cáceres-Rodríguez A, Strauss B, Chavis P, Manzoni OJ. Sexual differences in neuronal and synaptic properties across subregions of the mouse insular cortex. Biol Sex Differ. 2024;15(1):29. 38561860 10.1186/s13293-024-00593-4 PMC10983634

[63] Dai YJ, Zhang X, Yang Y, et al Gender differences in functional connectivities between insular subdivisions and selective pain-related brain structures. J Headache Pain. 2018;19(1):24. 29541875 10.1186/s10194-018-0849-z PMC5852124

[64] Galhardoni R, Aparecida da Silva V, García-Larrea L, et al Insular and anterior cingulate cortex deep stimulation for central neuropathic pain: Disassembling the percept of pain. Neurology. 2019;92(18):e2165–e2175. 30952795 10.1212/WNL.0000000000007396

[65] Dongyang L, Fernandes AM, da Cunha PHM, et al Posterior-superior insular deep transcranial magnetic stimulation alleviates peripheral neuropathic pain — A pilot double-blind, randomized cross-over study. Neurophysiol Clin. 2021;51(4):291–302. 34175192 10.1016/j.neucli.2021.06.003

[66] Lenoir C, Algoet M, Mouraux A. Deep continuous theta burst stimulation of the operculo-insular cortex selectively affects aδ-fibre heat pain. J Physiol. 2018;596(19):4767–4787. 30085357 10.1113/JP276359 PMC6166055

[67] Albanese MC, Duerden EG, Rainville P, Duncan GH. Memory traces of pain in human cortex. J Neurosci. 2007;27(17):4612–4620. 17460074 10.1523/JNEUROSCI.0695-07.2007 PMC6673011

[68] Bornhövd K, Quante M, Glauche V, Bromm B, Weiller C, Büchel C. Painful stimuli evoke different stimulus–response functions in the amygdala, prefrontal, insula and somatosensory cortex: A single-trial fMRI study. Brain. 2002;125(Pt 6):1326–1336. 12023321 10.1093/brain/awf137

[69] Cha M, Choi S, Kim K, Lee BH. Manganese-enhanced MRI depicts a reduction in brain responses to nociception upon mTOR inhibition in chronic pain rats. Mol Brain. 2020;13(1):158. 33267907 10.1186/s13041-020-00687-1 PMC7713325

[70] Henderson LA, Di Pietro F, Youssef AM, et al Effect of expectation on pain processing: A psychophysics and functional MRI analysis. Front Neurosci. 2020;14:6. 32082106 10.3389/fnins.2020.00006 PMC7004959

[71] Shi Y, Cui S, Zeng Y, et al Brain network to placebo and nocebo responses in acute experimental lower back pain: A multivariate granger causality analysis of fMRI data. Front Behav Neurosci. 2021;15:696577. 34566591 10.3389/fnbeh.2021.696577 PMC8458622

[72] Pierpaoli C, Basser PJ. Toward a quantitative assessment of diffusion anisotropy. Magn Reson Med. 1996;36(6):893–906. 8946355 10.1002/mrm.1910360612

[73] Čeko M, Shir Y, Ouellet JA, Ware MA, Stone LS, Seminowicz DA. Partial recovery of abnormal insula and dorsolateral prefrontal connectivity to cognitive networks in chronic low back pain after treatment. Hum Brain Mapp. 2015;36(6):2075–2092. 25648842 10.1002/hbm.22757 PMC6869701

[74] Huang X, Zhang D, Wang P, et al Altered amygdala effective connectivity in migraine without aura: Evidence from resting-state fMRI with granger causality analysis. J Headache Pain. 2021;22(1):25. 33858323 10.1186/s10194-021-01240-8 PMC8048057

[75] Tonini MC . Gender differences in migraine. Neurol Sci. 2018;39(Suppl 1):77–78. 29904873 10.1007/s10072-018-3378-2

[76] Li JN, Sheets PL. The central amygdala to periaqueductal gray pathway comprises intrinsically distinct neurons differentially affected in a model of inflammatory pain. J Physiol. 2018;596(24):6289–6305. 30281797 10.1113/JP276935 PMC6292805

[77] Floresco SB . The nucleus accumbens: An interface between cognition, emotion, and action. Annu Rev Psychol. 2015;66:25–52. 25251489 10.1146/annurev-psych-010213-115159

[78] Mitsi V, Zachariou V. Modulation of pain, nociception, and analgesia by the brain reward center. Neuroscience. 2016;338:81–92. 27189881 10.1016/j.neuroscience.2016.05.017 PMC5083150

[79] DosSantos MF, Moura BS, DaSilva AF. Reward circuitry plasticity in pain perception and modulation. Front Pharmacol. 2017;8:790. 29209204 10.3389/fphar.2017.00790 PMC5702349

[80] Gonzalez-Hermosillo DC, Gonzalez-Hermosillo LM, Villasenor-Almaraz M, et al Current concepts of pain pathways: A brief review of anatomy, physiology, and medical imaging. Curr Med Imaging. 2023;20. 10.2174/1573405620666230519144112 37211855

[81] Seminowicz DA, Remeniuk B, Krimmel SR, et al Pain-related nucleus accumbens function: Modulation by reward and sleep disruption. Pain. 2019;160(5):1196–1207. 30753171 10.1097/j.pain.0000000000001498 PMC7213641

[82] Harris HN, Peng YB. Evidence and explanation for the involvement of the nucleus accumbens in pain processing. Neural Regen Res. 2020;15(4):597–605. 31638081 10.4103/1673-5374.266909 PMC6975138

[83] Damascelli M, Woodward TS, Sanford N, et al Multiple functional brain networks related to pain perception revealed by fMRI. Neuroinformatics. 2022;20(1):155–172. 34101115 10.1007/s12021-021-09527-6 PMC9537130

[84] Journee SH, Mathis VP, Fillinger C, Veinante P, Yalcin I. Janus effect of the anterior cingulate cortex: Pain and emotion. Neurosci Biobehav Rev. 2023;153:105362. 37595650 10.1016/j.neubiorev.2023.105362

[85] Xiao X, Ding M, Zhang YQ. Role of the anterior cingulate Cortex in translational pain research. Neurosci Bull. 2021;37(3):405–422. 33566301 10.1007/s12264-020-00615-2 PMC7954910

[86] Singh A, Patel D, Li A, et al Mapping cortical integration of sensory and affective pain pathways. Curr Biol. 2020;30(9):1703–1715.e5. 32220320 10.1016/j.cub.2020.02.091 PMC7224326

[87] Xu Y, Lin Y, Yu M, Zhou K. The nucleus accumbens in reward and aversion processing: Insights and implications. Front Behav Neurosci. 2024;18:1420028. 39184934 10.3389/fnbeh.2024.1420028 PMC11341389

[88] Murray EA . The amygdala, reward and emotion. Trends Cogn Sci. 2007;11(11):489–497. 17988930 10.1016/j.tics.2007.08.013

[89] Friedman NP, Robbins TW. The role of prefrontal cortex in cognitive control and executive function. Neuropsychopharmacology. 2022;47(1):72–89. 34408280 10.1038/s41386-021-01132-0 PMC8617292

[90] Wang JY, Abdi H, Bakhadirov K, Diaz-Arrastia R, Devous MD Sr. A comprehensive reliability assessment of quantitative diffusion tensor tractography. Neuroimage. 2012;60(2):1127–1138. 22227883 10.1016/j.neuroimage.2011.12.062 PMC3468740

[91] Danielian LE, Iwata NK, Thomasson DM, Floeter MK. Reliability of fiber tracking measurements in diffusion tensor imaging for longitudinal study. NeuroImage. 2010;49(2):1572–1580. 19744567 10.1016/j.neuroimage.2009.08.062 PMC2789889

