# Supplementary material for: Sex differences in pain perception and modulation in the brain: effects of insular cortex stimulation on chronic pain relief
Source: Brain Commun. 2025 Sep 17;7(5):fcaf362. doi: 10.1093/braincomms/fcaf362 (PMC12492487; doi:10.1093/braincomms/fcaf362)
Supplement: fcaf362_Supplementary_Data [file fcaf362_supplementary_data.zip › Supplymentary Table 1 for Figure 3.pdf]

Sham	Male		Female		t	p	asterisk	Cohen's d
	n	Mean (±SEM)	n	Mean (±SEM)				
ACC-Amy	6	0.286 (±0.013)	9	0.300 (±0.009)	-0.917	0.381		0.499
ACC-IC	13	0.264 (±0.009)	15	0.279 (±0.008)	-1.301	0.205		0.491
ACC-NAcc	12	0.243 (±0.006)	13	0.272 (±0.008)	-2.896	0.008	**	1.149
ACC-PAG	13	0.254 (±0.006)	12	0.270 (±0.008)	-1.566	0.132		0.630
ACC-PFC	13	0.257 (±0.007)	15	0.279 (±0.010)	-1.8	0.084		0.664
ACC-S1	13	0.266 (±0.008)	14	0.278 (±0.009)	-0.934	0.359		0.358
ACC-S2	11	0.272 (±0.010)	14	0.292 (±0.010)	-1.39	0.178		0.547
ACC-VP	11	0.300 (±0.009)	14	0.304 (±0.008)	-0.38	0.708		0.154
Amy-IC	12	0.276 (±0.010)	14	0.283 (±0.009)	-0.516	0.611		0.204
Amy-NAcc	10	0.253 (±0.010)	7	0.289 (±0.008)	-2.75	0.015	*	1.258
Amy-PAG	12	0.269 (±0.006)	13	0.293 (±0.007)	-2.493	0.021	*	0.987
Amy-PFC	7	0.250 (±0.007)	8	0.287 (±0.012)	-2.652	0.022	*	1.327
Amy-S1	10	0.280 (±0.011)	10	0.288 (±0.011)	-0.522	0.608		0.233
Amy-S2	12	0.274 (±0.009)	12	0.285 (±0.008)	-0.897	0.380		0.366
Amy-VP	11	0.298 (±0.009)	12	0.310 (±0.007)	-1.045	0.309		0.439
IC-NAcc	13	0.234 (±0.008)	15	0.249 (±0.005)	-1.671	0.110		0.654
IC-PAG	13	0.264 (±0.012)	14	0.261 (±0.007)	0.257	0.800		0.101
IC-PFC	13	0.238 (±0.005)	15	0.243 (±0.007)	-0.556	0.584		0.205
IC-S1	12	0.256 (±0.014)	13	0.26 (±0.009)	-0.256	0.801		0.104
IC-S2	13	0.244 (±0.010)	15	0.254 (±0.009)	-0.777	0.445		0.296
IC-VP	12	0.261 (±0.011)	15	0.281 (±0.005)	-1.569	0.136		0.649
NAcc-PAG	13	0.249 (±0.007)	14	0.256 (±0.007)	-0.7	0.490		0.269
NAcc-PFC	13	0.242 (±0.008)	13	0.249 (±0.008)	-0.577	0.569		0.227
NAcc-S1	11	0.250 (±0.010)	9	0.254 (±0.012)	-0.21	0.836		0.095
NAcc-S2	4	0.243 (±0.015)	9	0.262 (±0.012)	-1.005	0.349		0.565
NAcc-VP	13	0.251 (±0.008)	15	0.277 (±0.008)	-2.23	0.035	*	0.840
PAG-PFC	9	0.251 (±0.011)	13	0.269 (±0.010)	-1.201	0.246		0.517
PAG-S1	10	0.263 (±0.009)	12	0.250 (±0.009)	0.979	0.340		0.417
PAG-S2	10	0.266 (±0.012)	9	0.287 (±0.010)	-1.314	0.207		0.596
PAG-VP	13	0.260 (±0.008)	15	0.284 (±0.008)	-2.124	0.043	*	0.796
PFC-S1	13	0.248 (±0.011)	13	0.260 (±0.007)	-0.938	0.360		0.368
PFC-S2	11	0.240 (±0.010)	14	0.259 (±0.013)	-1.151	0.262		0.442
PFC-VP	9	0.263 (±0.012)	11	0.298 (±0.007)	-2.627	0.020	*	1.230
S1-S2	12	0.256 (±0.013)	15	0.264 (±0.009)	-0.512	0.614		0.204
S1-VP	11	0.282 (±0.011)	13	0.280 (±0.006)	0.121	0.905		0.051
S2-VP	13	0.271 (±0.009)	15	0.291 (±0.009)	-1.543	0.135		0.585

NP	Male		Female		t	p	asterisk	Cohen's d
	n	Mean (±SEM)	n	Mean (±SEM)				
ACC-Amy	8	0.280 (±0.013)	5	0.312 (±0.022)	-1.23	0.259		0.747
ACC-IC	15	0.270 (±0.010)	16	0.263 (±0.007)	0.544	0.591		0.197
ACC-NAcc	15	0.241 (±0.009)	16	0.247 (±0.007)	-0.473	0.640		0.171
ACC-PAG	13	0.252 (±0.009)	15	0.255 (±0.005)	-0.291	0.774		0.115
ACC-PFC	16	0.265 (±0.009)	16	0.259 (±0.009)	0.542	0.591		0.192
ACC-S1	15	0.258 (±0.010)	16	0.259 (±0.009)	-0.125	0.901		0.045
ACC-S2	13	0.262 (±0.013)	15	0.257 (±0.011)	0.266	0.792		0.102
ACC-VP	12	0.258 (±0.010)	14	0.276 (±0.007)	-1.438	0.165		0.577
Amy-IC	12	0.252 (±0.008)	13	0.265 (±0.009)	-1.024	0.317		0.408
Amy-NAcc	11	0.255 (±0.008)	7	0.271 (±0.007)	-1.395	0.182		0.624
Amy-PAG	15	0.268 (±0.009)	15	0.286 (±0.007)	-1.534	0.137		0.56
Amy-PFC	7	0.257 (±0.011)	4	0.281 (±0.012)	-1.419	0.196		0.837
Amy-S1	7	0.265 (±0.011)	5	0.278 (±0.017)	-0.685	0.515		0.421
Amy-S2	12	0.262 (±0.011)	14	0.261 (±0.008)	0.02	0.985		0.008
Amy-VP	12	0.271 (±0.010)	12	0.309 (±0.009)	-2.811	0.010	*	1.147
IC-NAcc	16	0.238 (±0.008)	16	0.237 (±0.005)	0.149	0.883		0.053
IC-PAG	14	0.257 (±0.011)	14	0.256 (±0.007)	0.082	0.936		0.031
IC-PFC	16	0.243 (±0.009)	16	0.232 (±0.005)	1.057	0.301		0.374
IC-S1	13	0.232 (±0.010)	15	0.240 (±0.009)	-0.559	0.581		0.213
IC-S2	16	0.238 (±0.009)	16	0.229 (±0.007)	0.752	0.459		0.266
IC-VP	16	0.270 (±0.011)	16	0.268 (±0.009)	0.164	0.871		0.058
NAcc-PAG	12	0.244 (±0.008)	13	0.257 (±0.008)	-1.225	0.233		0.489
NAcc-PFC	14	0.237 (±0.008)	15	0.239 (±0.006)	-0.163	0.872		0.061
NAcc-S1	12	0.245 (±0.009)	13	0.247 (±0.008)	-0.185	0.855		0.074
NAcc-S2	13	0.238 (±0.008)	12	0.239 (±0.009)	-0.048	0.962		0.019
NAcc-VP	14	0.260 (±0.010)	16	0.264 (±0.008)	-0.366	0.717		0.135
PAG-PFC	11	0.250 (±0.012)	12	0.260 (±0.007)	-0.727	0.478		0.31
PAG-S1	10	0.258 (±0.012)	13	0.256 (±0.006)	0.17	0.867		0.076
PAG-S2	13	0.259 (±0.012)	13	0.257 (±0.006)	0.112	0.912		0.044
PAG-VP	16	0.261 (±0.010)	14	0.256 (±0.005)	0.398	0.694		0.14
PFC-S1	14	0.239 (±0.011)	14	0.244 (±0.011)	-0.291	0.774		0.11
PFC-S2	15	0.241 (±0.011)	14	0.236 (±0.013)	0.311	0.758		0.116
PFC-VP	11	0.265 (±0.011)	11	0.272 (±0.009)	-0.476	0.639		0.203
S1-S2	14	0.232 (±0.009)	13	0.232 (±0.006)	0.05	0.960		0.019
S1-VP	11	0.271 (±0.014)	15	0.264 (±0.008)	0.429	0.673		0.18
S2-VP	14	0.270 (±0.013)	15	0.284 (±0.011)	-0.841	0.408		0.314

ICS	Male		Female		t	p	asterisk	Cohen's d
	n	Mean (±SEM)	n	Mean (±SEM)				
ACC-Amy	6	0.27 (±0.016)	8	0.291 (±0.014)	-0.945	0.366		0.513
ACC-IC	17	0.297 (±0.008)	16	0.273 (±0.008)	1.982	0.056		0.69
ACC-NAcc	17	0.26 (±0.007)	14	0.244 (±0.007)	1.591	0.123		0.566
ACC-PAG	15	0.265 (±0.007)	13	0.263 (±0.005)	0.262	0.795		0.097
ACC-PFC	17	0.287 (±0.006)	16	0.269 (±0.007)	1.862	0.073		0.653
ACC-S1	17	0.27 (±0.005)	15	0.258 (±0.006)	1.564	0.129		0.558
ACC-S2	13	0.281 (±0.008)	15	0.261 (±0.01)	1.562	0.130		0.582
ACC-VP	14	0.305 (±0.009)	15	0.28 (±0.006)	2.303	0.030	*	0.865
Amy-IC	13	0.269 (±0.008)	15	0.269 (±0.006)	-0.017	0.986		0.007
Amy-NAcc	10	0.26 (±0.009)	8	0.277 (±0.009)	-1.301	0.212		0.61
Amy-PAG	14	0.283 (±0.01)	14	0.289 (±0.007)	-0.518	0.610		0.196
Amy-PFC	4	0.263 (±0.012)	7	0.278 (±0.018)	-0.722	0.488		0.381
Amy-S1	6	0.268 (±0.017)	6	0.28 (±0.013)	-0.561	0.588		0.324
Amy-S2	10	0.268 (±0.01)	12	0.27 (±0.009)	-0.182	0.858		0.078
Amy-VP	14	0.302 (±0.009)	15	0.304 (±0.005)	-0.174	0.864		0.066
IC-NAcc	18	0.251 (±0.006)	16	0.247 (±0.007)	0.534	0.597		0.184
IC-PAG	16	0.27 (±0.008)	15	0.261 (±0.005)	0.975	0.339		0.346
IC-PFC	18	0.261 (±0.01)	16	0.243 (±0.007)	1.466	0.153		0.494
IC-S1	15	0.272 (±0.008)	14	0.236 (±0.006)	3.577	0.001	**	1.31
IC-S2	18	0.246 (±0.006)	14	0.243 (±0.007)	0.338	0.738		0.12
IC-VP	18	0.289 (±0.007)	15	0.264 (±0.005)	2.864	0.008	**	0.963
NAcc-PAG	14	0.262 (±0.008)	14	0.256 (±0.006)	0.634	0.533		0.24
NAcc-PFC	16	0.253 (±0.01)	15	0.248 (±0.009)	0.381	0.706		0.136
NAcc-S1	7	0.271 (±0.014)	7	0.265 (±0.013)	0.318	0.756		0.17
NAcc-S2	13	0.255 (±0.011)	15	0.243 (±0.008)	0.84	0.410		0.324
NAcc-VP	16	0.295 (±0.007)	15	0.268 (±0.005)	2.966	0.006	**	1.055
PAG-PFC	11	0.268 (±0.009)	10	0.26 (±0.011)	0.553	0.587		0.243
PAG-S1	11	0.259 (±0.008)	11	0.256 (±0.006)	0.239	0.814		0.102
PAG-S2	13	0.281 (±0.01)	12	0.256 (±0.007)	2.085	0.050		0.819
PAG-VP	18	0.277 (±0.009)	16	0.273 (±0.008)	0.38	0.707		0.129
PFC-S1	14	0.266 (±0.01)	14	0.241 (±0.006)	2.255	0.035	*	0.852
PFC-S2	17	0.25 (±0.01)	14	0.259 (±0.011)	-0.6	0.553		0.217
PFC-VP	13	0.269 (±0.011)	10	0.266 (±0.004)	0.26	0.798		0.098
S1-S2	13	0.246 (±0.009)	14	0.244 (±0.006)	0.199	0.844		0.078
S1-VP	12	0.297 (±0.007)	15	0.274 (±0.006)	2.531	0.019	*	0.996
S2-VP	18	0.285 (±0.009)	16	0.267 (±0.006)	1.604	0.119		0.538

Supplymentary Table 1 for Figure 3. FA value comparisons across pain-related brain regions.
