# Supplementary material for: Sex differences in pain perception and modulation in the brain: effects of insular cortex stimulation on chronic pain relief
Source: Brain Commun. 2025 Sep 17;7(5):fcaf362. doi: 10.1093/braincomms/fcaf362 (PMC12492487; doi:10.1093/braincomms/fcaf362)
Supplement: fcaf362_Supplementary_Data [file fcaf362_supplementary_data.zip › Supplymentary Table 2 for Figure 3.pdf]

Sham.1	Male.3	Female.3	SEM_M	SEM_F	nSHM	nSHF
ACC-Amy	0.28567	0.30021	0.012966	0.009118	6	9
ACC-IC	0.26378	0.27944	0.008589	0.008433	13	15
ACC-NAcc	0.24294	0.27153	0.006336	0.007567	12	13
ACC-PAG	0.25394	0.26958	0.006392	0.007681	13	12
ACC-PFC	0.25737	0.27906	0.007175	0.009684	13	15
ACC-S1	0.26612	0.27787	0.008415	0.009353	13	14
ACC-S2	0.27224	0.29208	0.009759	0.010411	11	14
ACC-VP	0.29974	0.30442	0.009428	0.007922	11	14
Amy-IC	0.27577	0.28263	0.010083	0.008679	12	14
Amy-NAcc	0.2534	0.28885	0.010131	0.007969	10	7
Amy-PAG	0.26908	0.29274	0.005861	0.00746	12	13
Amy-PFC	0.25032	0.28702	0.007477	0.011642	7	8
Amy-S1	0.27969	0.28781	0.010758	0.011224	10	10
Amy-S2	0.27411	0.28503	0.009045	0.008159	12	12
Amy-VP	0.29828	0.31034	0.008915	0.007339	11	12
IC-NAcc	0.23353	0.24908	0.008011	0.004728	13	15
IC-PAG	0.26445	0.26088	0.01201	0.006955	13	14
IC-PFC	0.23824	0.243	0.004976	0.00699	13	15
IC-S1	0.25552	0.25975	0.013772	0.009184	12	13
IC-S2	0.2437	0.2543	0.010443	0.008799	13	15
IC-VP	0.26128	0.28072	0.011216	0.005255	12	15
NAcc-PAG	0.24871	0.25595	0.007287	0.007342	13	14
NAcc-PFC	0.24249	0.24904	0.007591	0.008429	13	13
NAcc-S1	0.25024	0.2535	0.010046	0.011854	11	9
NAcc-S2	0.24318	0.26222	0.014921	0.01167	4	9
NAcc-VP	0.25131	0.27734	0.008217	0.008287	13	15
PAG-PFC	0.2511	0.26871	0.0111	0.009577	9	13
PAG-S1	0.26276	0.24996	0.009468	0.009022	10	12
PAG-S2	0.26637	0.28675	0.011972	0.009869	10	9
PAG-VP	0.26014	0.28378	0.007524	0.008197	13	15
PFC-S1	0.24758	0.26005	0.011385	0.006856	13	13
PFC-S2	0.23963	0.25879	0.009989	0.013322	11	14
PFC-VP	0.26256	0.29802	0.01157	0.006948	9	11
S1-S2	0.25607	0.26435	0.013311	0.009171	12	15
S1-VP	0.28202	0.28047	0.011064	0.006436	11	13
S2-VP	0.27129	0.29126	0.00946	0.008827	13	15

NP.1	Male.4	Female.4	SEM_M.1	SEM_F.1	nNPM	nNPF
ACC-Amy	0.28016	0.31187	0.013451	0.022007	8	5
ACC-IC	0.2698	0.26328	0.009538	0.007269	15	16
ACC-NAcc	0.24133	0.24654	0.008604	0.006854	15	16
ACC-PAG	0.25179	0.25489	0.009465	0.004881	13	15
ACC-PFC	0.26536	0.2587	0.008701	0.008671	16	16
ACC-S1	0.25767	0.25931	0.009864	0.008585	15	16
ACC-S2	0.26175	0.25715	0.013475	0.010843	13	15
ACC-VP	0.25798	0.27597	0.010141	0.007323	12	14
Amy-IC	0.25234	0.26451	0.00809	0.008713	12	13
Amy-NAcc	0.25512	0.27074	0.008374	0.007432	11	7
Amy-PAG	0.2681	0.28567	0.008841	0.007276	15	15
Amy-PFC	0.25704	0.28093	0.011447	0.01235	7	4
Amy-S1	0.2649	0.27841	0.010647	0.016602	7	5
Amy-S2	0.26154	0.26127	0.010749	0.008354	12	14
Amy-VP	0.27083	0.30918	0.00989	0.009403	12	12
IC-NAcc	0.23828	0.23685	0.008026	0.005199	16	16
IC-PAG	0.25682	0.25572	0.011465	0.006993	14	14
IC-PFC	0.24305	0.2324	0.0085	0.00542	16	16
IC-S1	0.23244	0.24018	0.010381	0.009148	13	15
IC-S2	0.23817	0.22926	0.009452	0.007147	16	16
IC-VP	0.27036	0.26811	0.010603	0.008744	16	16
NAcc-PAG	0.24366	0.25734	0.007881	0.007922	12	13
NAcc-PFC	0.23731	0.23886	0.007501	0.005852	14	15
NAcc-S1	0.2445	0.24672	0.008738	0.00828	12	13
NAcc-S2	0.23797	0.23858	0.008237	0.009455	13	12
NAcc-VP	0.25976	0.26439	0.009863	0.007909	14	16
PAG-PFC	0.24979	0.25974	0.011955	0.006654	11	12
PAG-S1	0.25777	0.2555	0.011745	0.006385	10	13
PAG-S2	0.25884	0.25734	0.01182	0.006142	13	13
PAG-VP	0.26073	0.25643	0.009604	0.004944	16	14
PFC-S1	0.23947	0.24395	0.010561	0.011248	14	14
PFC-S2	0.24104	0.2358	0.010581	0.013112	15	14
PFC-VP	0.26526	0.27207	0.010917	0.00924	11	11
S1-S2	0.23248	0.23197	0.008511	0.00572	14	13
S1-VP	0.27126	0.26433	0.01383	0.008301	11	15
S2-VP	0.26966	0.28352	0.012663	0.010544	14	15

ICS.1	Male.5	Female.5	SEM_M.2	SEM_F.2	nICSM	nICSF
ACC-Amy	0.27048	0.29074	0.016446	0.013761	6	8
ACC-IC	0.29655	0.27326	0.008178	0.008434	17	16
ACC-NAcc	0.26041	0.24395	0.007467	0.007169	17	14
ACC-PAG	0.26477	0.26253	0.006851	0.005151	15	13
ACC-PFC	0.28654	0.26897	0.005778	0.007459	17	16
ACC-S1	0.27006	0.25791	0.004961	0.005975	17	15
ACC-S2	0.28087	0.26073	0.008351	0.009826	13	15
ACC-VP	0.30494	0.27972	0.008958	0.006297	14	15
Amy-IC	0.26893	0.26909	0.00756	0.005647	13	15
Amy-NAcc	0.26015	0.27719	0.009197	0.009334	10	8
Amy-PAG	0.28257	0.28873	0.009914	0.006592	14	14
Amy-PFC	0.26296	0.27846	0.012167	0.017681	4	7
Amy-S1	0.26755	0.27956	0.016716	0.013395	6	6
Amy-S2	0.26801	0.27042	0.01009	0.00857	10	12
Amy-VP	0.3023	0.30402	0.008583	0.004922	14	15
IC-NAcc	0.25134	0.2465	0.005965	0.006805	18	16
IC-PAG	0.27009	0.26097	0.007738	0.005268	16	15
IC-PFC	0.2609	0.24322	0.009597	0.007303	18	16
IC-S1	0.27151	0.23613	0.008182	0.005555	15	14
IC-S2	0.24574	0.24253	0.006458	0.006924	18	14
IC-VP	0.28946	0.26414	0.007243	0.00507	18	15
NAcc-PAG	0.26249	0.2562	0.008232	0.005569	14	14
NAcc-PFC	0.25338	0.24847	0.009539	0.00867	16	15
NAcc-S1	0.27102	0.26504	0.013659	0.012921	7	7
NAcc-S2	0.25476	0.24349	0.010826	0.007926	13	15
NAcc-VP	0.29497	0.26796	0.007275	0.005475	16	15
PAG-PFC	0.26772	0.26006	0.008842	0.010644	11	10
PAG-S1	0.2588	0.25644	0.007799	0.006115	11	11
PAG-S2	0.28111	0.2556	0.010311	0.006593	13	12
PAG-VP	0.27702	0.27259	0.008565	0.007894	18	16
PFC-S1	0.26638	0.24123	0.009585	0.005703	14	14
PFC-S2	0.25019	0.25925	0.010105	0.011229	17	14
PFC-VP	0.26911	0.26593	0.011359	0.004428	13	10
S1-S2	0.24635	0.24428	0.008751	0.005646	13	14
S1-VP	0.29715	0.2744	0.007096	0.00552	12	15
S2-VP	0.28477	0.26693	0.009132	0.006354	18	16

tract	Original p	FDR-corrected p	Cohen's d (final)
ACC-Amy	0.381	0.5715	0.499
ACC-IC	0.205	0.4968	0.491
ACC-NAcc	0.008	0.1584	1.149
ACC-PAG	0.132	0.408	0.63
ACC-PFC	0.084	0.378	0.664
ACC-S1	0.359	0.5715	0.358
ACC-S2	0.178	0.492923077	0.547
ACC-VP	0.708	0.7965	0.154
Amy-IC	0.611	0.713032258	0.204
Amy-NAcc	0.015	0.1584	1.258
Amy-PAG	0.021	0.1584	0.987
Amy-PFC	0.022	0.1584	1.327
Amy-S1	0.608	0.713032258	0.233
Amy-S2	0.38	0.5715	0.366
Amy-VP	0.309	0.5715	0.439
IC-NAcc	0.11	0.408	0.654
IC-PAG	0.8	0.848117647	0.101
IC-PFC	0.584	0.713032258	0.205
IC-S1	0.801	0.848117647	0.104
IC-S2	0.445	0.6408	0.296
IC-VP	0.136	0.408	0.649
NAcc-PAG	0.49	0.678461538	0.269
NAcc-PFC	0.569	0.713032258	0.227
NAcc-S1	0.836	0.859885714	0.095
NAcc-S2	0.349	0.5715	0.565
NAcc-VP	0.035	0.21	0.84
PAG-PFC	0.246	0.5535	0.517
PAG-S1	0.34	0.5715	0.417
PAG-S2	0.207	0.4968	0.596
PAG-VP	0.043	0.221142857	0.796
PFC-S1	0.36	0.5715	0.368
PFC-S2	0.262	0.554823529	0.442
PFC-VP	0.02	0.1584	1.23
S1-S2	0.614	0.713032258	0.204
S1-VP	0.905	0.905	0.051
S2-VP	0.135	0.408	0.585

Supplymentary Table 2 for Figure 3. Comparison of FDR-corrected p and Cohen's d valuses across pain-related brain regions
