# Supplementary material for: Sex differences in pain perception and modulation in the brain: effects of insular cortex stimulation on chronic pain relief
Source: Brain Commun. 2025 Sep 17;7(5):fcaf362. doi: 10.1093/braincomms/fcaf362 (PMC12492487; doi:10.1093/braincomms/fcaf362)
Supplement: fcaf362_Supplementary_Data [file fcaf362_supplementary_data.zip › Supplymentary Table 3 for Figure 4.pdf]

Path	Sham_M	Sham_F	NP_M	NP_F	ICS_M	ICS_F	nSham_M	nSham_F	nNP_M	nNP_F	nICS_M	nICS_F	tSH:M-F	pSH:M-F	tNP:M-F	pNP:M-F	tICS:M-F	pICS:M-F
**fa_ac_am**	0.286	0.300	0.280	0.312	0.270	0.291	0.683	0.859	0.616	1.000	0.499	0.744	-0.917	0.381	-1.230	0.259	-0.945	0.366
**fa_ac_ic**	0.264	0.279	0.270	0.263	0.297	0.273	0.418	0.607	0.491	0.412	0.815	0.533	-1.301	0.205	0.544	0.591	1.982	0.056
**fa_ac_na**	0.243	0.272	0.241	0.247	0.260	0.244	0.166	0.512	0.146	0.209	0.377	0.178	-2.896	0.008	-0.473	0.640	1.591	0.123
**fa_ac_pa**	0.254	0.270	0.252	0.255	0.265	0.263	0.299	0.488	0.273	0.310	0.430	0.403	-1.566	0.132	-0.291	0.774	0.262	0.795
**fa_ac_pf**	0.257	0.279	0.265	0.259	0.287	0.269	0.340	0.603	0.437	0.356	0.693	0.481	-1.800	0.084	0.542	0.591	1.862	0.073
**fa_ac_s1**	0.266	0.278	0.258	0.259	0.270	0.258	0.446	0.588	0.344	0.364	0.494	0.347	-0.934	0.359	-0.125	0.901	1.564	0.129
**fa_ac_s2**	0.272	0.292	0.262	0.257	0.281	0.261	0.520	0.760	0.393	0.338	0.625	0.381	-1.390	0.178	0.266	0.792	1.562	0.130
**fa_ac_vp**	0.300	0.304	0.258	0.276	0.305	0.280	0.853	0.910	0.348	0.565	0.916	0.611	-0.380	0.708	-1.438	0.165	2.303	0.030
**fa_am_ic**	0.276	0.283	0.252	0.265	0.269	0.269	0.563	0.646	0.279	0.427	0.480	0.482	-0.516	0.611	-1.024	0.317	-0.017	0.986
**fa_am_na**	0.253	0.289	0.255	0.271	0.260	0.277	0.292	0.721	0.313	0.502	0.374	0.580	-2.750	0.015	-1.395	0.182	-1.301	0.212
**fa_am_pa**	0.269	0.293	0.268	0.286	0.283	0.289	0.482	0.768	0.470	0.683	0.645	0.720	-2.493	0.021	-1.534	0.137	-0.518	0.610
**fa_am_pf**	0.250	0.287	0.257	0.281	0.263	0.278	0.255	0.699	0.336	0.625	0.408	0.596	-2.652	0.022	-1.419	0.196	-0.722	0.488
**fa_am_s1**	0.280	0.288	0.265	0.278	0.268	0.280	0.610	0.709	0.431	0.595	0.464	0.609	-0.522	0.608	-0.685	0.515	-0.561	0.588
**fa_am_s2**	0.274	0.285	0.262	0.261	0.268	0.270	0.543	0.675	0.391	0.387	0.469	0.498	-0.897	0.380	0.020	0.985	-0.182	0.858
**fa_am_vp**	0.298	0.310	0.271	0.309	0.302	0.304	0.835	0.981	0.503	0.967	0.884	0.905	-1.045	0.309	-2.811	0.010	-0.174	0.864
**fa_ic_na**	0.234	0.249	0.238	0.237	0.251	0.247	0.052	0.240	0.109	0.092	0.267	0.209	-1.671	0.110	0.149	0.883	0.534	0.597
**fa_ic_pa**	0.264	0.261	0.257	0.256	0.270	0.261	0.426	0.383	0.334	0.320	0.494	0.384	0.257	0.800	0.082	0.936	0.975	0.339
**fa_ic_pf**	0.238	0.243	0.243	0.232	0.261	0.243	0.109	0.166	0.167	0.038	0.383	0.169	-0.556	0.584	1.057	0.301	1.466	0.153
**fa_ic_s1**	0.256	0.260	0.232	0.240	0.272	0.236	0.318	0.369	0.038	0.132	0.511	0.083	-0.256	0.801	-0.559	0.581	3.577	0.001
**fa_ic_s2**	0.244	0.254	0.238	0.229	0.246	0.243	0.175	0.303	0.108	0.000	0.199	0.161	-0.777	0.445	0.752	0.459	0.338	0.738
**fa_ic_vp**	0.261	0.281	0.270	0.268	0.289	0.264	0.388	0.623	0.498	0.470	0.729	0.422	-1.569	0.136	0.164	0.871	2.864	0.008
**fa_na_pa**	0.249	0.256	0.244	0.257	0.262	0.256	0.235	0.323	0.174	0.340	0.402	0.326	-0.700	0.490	-1.225	0.233	0.634	0.533
**fa_na_pf**	0.242	0.249	0.237	0.239	0.253	0.248	0.160	0.239	0.097	0.116	0.292	0.233	-0.577	0.569	-0.163	0.872	0.381	0.706
**fa_na_s1**	0.250	0.254	0.245	0.247	0.271	0.265	0.254	0.293	0.184	0.211	0.506	0.433	-0.210	0.836	-0.185	0.855	0.318	0.756
**fa_na_s2**	0.243	0.262	0.238	0.239	0.255	0.243	0.169	0.399	0.105	0.113	0.309	0.172	-1.005	0.349	-0.048	0.962	0.840	0.410
**fa_na_vp**	0.251	0.277	0.260	0.264	0.295	0.268	0.267	0.582	0.369	0.425	0.795	0.468	-2.230	0.035	-0.366	0.717	2.966	0.006
**fa_pa_pf**	0.251	0.269	0.250	0.260	0.268	0.260	0.264	0.478	0.249	0.369	0.466	0.373	-1.201	0.246	-0.727	0.478	0.553	0.587
**fa_pa_s1**	0.263	0.250	0.258	0.256	0.259	0.256	0.406	0.251	0.345	0.318	0.358	0.329	0.979	0.340	0.170	0.867	0.239	0.814
**fa_pa_s2**	0.266	0.287	0.259	0.257	0.281	0.256	0.449	0.696	0.358	0.340	0.628	0.319	-1.314	0.207	0.112	0.912	2.085	0.050
**fa_pa_vp**	0.260	0.284	0.261	0.256	0.277	0.273	0.374	0.660	0.381	0.329	0.578	0.525	-2.124	0.043	0.398	0.694	0.380	0.707
**fa_pf_s1**	0.248	0.260	0.239	0.244	0.266	0.241	0.222	0.373	0.124	0.178	0.449	0.145	-0.938	0.360	-0.291	0.774	2.255	0.035
**fa_pf_s2**	0.240	0.259	0.241	0.236	0.250	0.259	0.126	0.357	0.143	0.079	0.253	0.363	-1.151	0.262	0.311	0.758	-0.600	0.553
**fa_pf_vp**	0.263	0.298	0.265	0.272	0.269	0.266	0.403	0.832	0.436	0.518	0.482	0.444	-2.627	0.020	-0.476	0.639	0.260	0.798
**fa_s1_s2**	0.256	0.264	0.232	0.232	0.246	0.244	0.325	0.425	0.039	0.033	0.207	0.182	-0.512	0.614	0.050	0.960	0.199	0.844
**fa_s1_vp**	0.282	0.280	0.271	0.264	0.297	0.274	0.639	0.620	0.508	0.425	0.822	0.546	0.121	0.905	0.429	0.673	2.531	0.019
**fa_s2_vp**	0.271	0.291	0.270	0.284	0.285	0.267	0.509	0.751	0.489	0.657	0.672	0.456	-1.543	0.135	-0.841	0.408	1.604	0.119

Supplymentary Table 3 for Figure 4. Quantification of FA values for sham, NP, and ICS male/female groups
