# Supplementary material for: Sex differences in pain perception and modulation in the brain: effects of insular cortex stimulation on chronic pain relief
Source: Brain Commun. 2025 Sep 17;7(5):fcaf362. doi: 10.1093/braincomms/fcaf362 (PMC12492487; doi:10.1093/braincomms/fcaf362)
Supplement: fcaf362_Supplementary_Data [file fcaf362_supplementary_data.zip › Supplymentary Table 4 for Figure 5.pdf]

Male	Sham	NP	ICS	ANOVA			Tukey's HSD			
				F	p-value	asterisk	SHvsNP	SHvsICS	NPvsICS	Subset
ACC-Amy	0.286 (±0.013)	0.280 (±0.013)	0.270 (±0.016)	0.260	0.774					
ACC-IC	0.264 (±0.009)	0.270 (±0.010)	0.297 (±0.008)	4.082	0.024	*		0.033		Sham<ICS
ACC-NAcc	0.243 (±0.006)	0.241 (±0.009)	0.260 (±0.007)	2.013	0.147					
ACC-PAG	0.254 (±0.006)	0.252 (±0.009)	0.265 (±0.007)	0.867	0.428					
ACC-PFC	0.257 (±0.007)	0.265 (±0.009)	0.287 (±0.006)	4.283	0.020	*		0.022		Sham<ICS
ACC-S1	0.266 (±0.008)	0.258 (±0.010)	0.270 (±0.005)	0.691	0.507					
ACC-S2	0.272 (±0.010)	0.262 (±0.013)	0.281 (±0.008)	0.815	0.451					
ACC-VP	0.300 (±0.009)	0.258 (±0.010)	0.305 (±0.009)	7.320	0.002	**	0.014		0.003	NP<Sham,ICS
Amy-IC	0.276 (±0.010)	0.252 (±0.008)	0.269 (±0.008)	1.918	0.162					
Amy-NAcc	0.253 (±0.010)	0.255 (±0.008)	0.260 (±0.009)	0.141	0.869					
Amy-PAG	0.269 (±0.006)	0.268 (±0.009)	0.283 (±0.010)	0.891	0.419					
Amy-PFC	0.250 (±0.007)	0.257 (±0.011)	0.263 (±0.012)	0.332	0.723					
Amy-S1	0.280 (±0.011)	0.265 (±0.011)	0.268 (±0.017)	0.451	0.643					
Amy-S2	0.274 (±0.009)	0.262 (±0.011)	0.268 (±0.010)	0.417	0.662					
Amy-VP	0.298 (±0.009)	0.271 (±0.010)	0.302 (±0.009)	3.518	0.041	*			0.046	NP<ICS
IC-NAcc	0.234 (±0.008)	0.238 (±0.008)	0.251 (±0.006)	1.648	0.204					
IC-PAG	0.264 (±0.012)	0.257 (±0.011)	0.270 (±0.008)	0.434	0.651					
IC-PFC	0.238 (±0.005)	0.243 (±0.009)	0.261 (±0.010)	2.054	0.140					
IC-S1	0.256 (±0.014)	0.232 (±0.010)	0.272 (±0.008)	3.531	0.040	*			0.031	NP<ICS
IC-S2	0.244 (±0.010)	0.238 (±0.009)	0.246 (±0.006)	0.218	0.805					
IC-VP	0.261 (±0.011)	0.270 (±0.011)	0.289 (±0.007)	2.292	0.113					
NAcc-PAG	0.249 (±0.007)	0.244 (±0.008)	0.262 (±0.008)	1.573	0.221					
NAcc-PFC	0.242 (±0.008)	0.237 (±0.008)	0.253 (±0.010)	0.989	0.381					
NAcc-S1	0.250 (±0.010)	0.245 (±0.009)	0.271 (±0.014)	1.500	0.241					
NAcc-S2	0.243 (±0.015)	0.238 (±0.008)	0.255 (±0.011)	0.799	0.460					
NAcc-VP	0.251 (±0.008)	0.260 (±0.010)	0.295 (±0.007)	7.808	0.001	**		0.002	0.012	Sham,NP<ICS
PAG-PFC	0.251 (±0.011)	0.250 (±0.012)	0.268 (±0.009)	0.903	0.417					
PAG-S1	0.263 (±0.009)	0.258 (±0.012)	0.259 (±0.008)	0.072	0.931					
PAG-S2	0.266 (±0.012)	0.259 (±0.012)	0.281 (±0.010)	1.068	0.355					
PAG-VP	0.260 (±0.008)	0.261 (±0.010)	0.277 (±0.009)	1.254	0.295					
PFC-S1	0.248 (±0.011)	0.239 (±0.011)	0.266 (±0.010)	1.767	0.185					
PFC-S2	0.240 (±0.010)	0.241 (±0.011)	0.250 (±0.010)	0.318	0.730					
PFC-VP	0.263 (±0.012)	0.265 (±0.011)	0.269 (±0.011)	0.083	0.920					
S1-S2	0.256 (±0.013)	0.232 (±0.009)	0.246 (±0.009)	1.363	0.269					
S1-VP	0.282 (±0.011)	0.271 (±0.014)	0.297 (±0.007)	1.470	0.246					
S2-VP	0.271 (±0.009)	0.270 (±0.013)	0.285 (±0.009)	0.683	0.510					

Female	Sham	NP	ICS	ANOVA			Tukey's HSD			
				F	p-value	asterisk	SHvsNP	SHvsICS	NPvsICS	Subset
ACC-Amy	0.300 (±0.009)	0.312 (±0.022)	0.291 (±0.014)	0.500	0.614					
ACC-IC	0.279 (±0.008)	0.263 (±0.007)	0.273 (±0.008)	1.020	0.369					
ACC-NAcc	0.272 (±0.008)	0.247 (±0.007)	0.244 (±0.007)	4.247	0.021	*	0.047	0.031		ICS,NP<Sham
ACC-PAG	0.270 (±0.008)	0.255 (±0.005)	0.263 (±0.005)	1.584	0.219					
ACC-PFC	0.279 (±0.010)	0.259 (±0.009)	0.269 (±0.007)	1.383	0.262					
ACC-S1	0.278 (±0.009)	0.259 (±0.009)	0.258 (±0.006)	1.832	0.173					
ACC-S2	0.292 (±0.010)	0.257 (±0.011)	0.261 (±0.010)	3.353	0.045	*				
ACC-VP	0.304 (±0.008)	0.276 (±0.007)	0.280 (±0.006)	4.567	0.016	*	0.022	0.048		NP,ICS<Sham
Amy-IC	0.283 (±0.009)	0.265 (±0.009)	0.269 (±0.006)	1.473	0.242					
Amy-NAcc	0.289 (±0.008)	0.271 (±0.007)	0.277 (±0.009)	1.139	0.341					
Amy-PAG	0.293 (±0.007)	0.286 (±0.007)	0.289 (±0.007)	0.245	0.784					
Amy-PFC	0.287 (±0.012)	0.281 (±0.012)	0.278 (±0.018)	0.102	0.904					
Amy-S1	0.288 (±0.011)	0.278 (±0.017)	0.280 (±0.013)	0.165	0.850					
Amy-S2	0.285 (±0.008)	0.261 (±0.008)	0.270 (±0.009)	2.063	0.142					
Amy-VP	0.310 (±0.007)	0.309 (±0.009)	0.304 (±0.005)	0.236	0.791					
IC-NAcc	0.249 (±0.005)	0.237 (±0.005)	0.247 (±0.007)	1.287	0.286					
IC-PAG	0.261 (±0.007)	0.256 (±0.007)	0.261 (±0.005)	0.217	0.806					
IC-PFC	0.243 (±0.007)	0.232 (±0.005)	0.243 (±0.007)	0.886	0.420					
IC-S1	0.26 (±0.009)	0.24 (±0.009)	0.236 (±0.006)	2.294	0.114					
IC-S2	0.254 (±0.009)	0.229 (±0.007)	0.243 (±0.007)	2.745	0.076					
IC-VP	0.281 (±0.005)	0.268 (±0.009)	0.264 (±0.005)	1.642	0.206					
NAcc-PAG	0.256 (±0.007)	0.257 (±0.008)	0.256 (±0.006)	0.011	0.989					
NAcc-PFC	0.249 (±0.008)	0.239 (±0.006)	0.248 (±0.009)	0.560	0.575					
NAcc-S1	0.254 (±0.012)	0.247 (±0.008)	0.265 (±0.013)	0.714	0.499					
NAcc-S2	0.262 (±0.012)	0.239 (±0.009)	0.243 (±0.008)	1.477	0.243					
NAcc-VP	0.277 (±0.008)	0.264 (±0.008)	0.268 (±0.005)	0.825	0.445					
PAG-PFC	0.269 (±0.010)	0.26 (±0.007)	0.260 (±0.011)	0.336	0.717					
PAG-S1	0.250 (±0.009)	0.256 (±0.006)	0.256 (±0.006)	0.226	0.799					
PAG-S2	0.287 (±0.010)	0.257 (±0.006)	0.256 (±0.007)	5.052	0.013	*	0.025	0.019		ICS,NP<Sham
PAG-VP	0.284 (±0.008)	0.256 (±0.005)	0.273 (±0.008)	3.420	0.042	*	0.033			NP<Sham
PFC-S1	0.260 (±0.007)	0.244 (±0.011)	0.241 (±0.006)	1.444	0.249					
PFC-S2	0.259 (±0.013)	0.236 (±0.013)	0.259 (±0.011)	1.134	0.332					
PFC-VP	0.298 (±0.007)	0.272 (±0.009)	0.266 (±0.004)	5.474	0.010	*	0.041	0.012		ICS,NP<Sham
S1-S2	0.264 (±0.009)	0.232 (±0.006)	0.244 (±0.006)	5.146	0.010	*	0.008			NP<Sham
S1-VP	0.280 (±0.006)	0.264 (±0.008)	0.274 (±0.006)	1.375	0.265					
S2-VP	0.291 (±0.009)	0.284 (±0.011)	0.267 (±0.006)	2.097	0.135					

Supplymentary Table 4 for Figure 5. Comparison of FA values in functional connectivity among Sham, NP and ICS groups
