# Supplementary material for: Sex differences in pain perception and modulation in the brain: effects of insular cortex stimulation on chronic pain relief
Source: Brain Commun. 2025 Sep 17;7(5):fcaf362. doi: 10.1093/braincomms/fcaf362 (PMC12492487; doi:10.1093/braincomms/fcaf362)
Supplement: fcaf362_Supplementary_Data [file fcaf362_supplementary_data.zip › Supplymentary Table 5 for Figure 6.pdf]

Path	Sham_M	Sham_F	NP_M	NP_F	ICS_M	ICS_F	tM:NP-SH	tF:NP-SH	tM:IC-SH	tF:IC-SH	tM:IC-NP	tF:IC-NP	tM:NP-ICS	tF:NP-ICS
**fa_ac_am**	0.28567	0.30021	0.28016	0.31187	0.27048	0.29074	-0.2950	0.4900	-0.7250	-0.5730	-0.4560	-0.8140	0.4560	0.8140
**fa_ac_ic**	0.26378	0.27944	0.2698	0.26328	0.29655	0.27326	0.4690	-1.4520	2.7630	-0.5180	2.1290	0.8970	-2.1290	-0.8970
**fa_ac_na**	0.24294	0.27153	0.24133	0.24654	0.26041	0.24395	-0.1510	-2.4480	1.7840	-2.6460	1.6750	-0.2610	-1.6750	0.2610
**fa_ac_pa**	0.25394	0.26958	0.25179	0.25489	0.26477	0.26253	-0.1880	-1.6140	1.1560	-0.7630	1.1110	1.0760	-1.1110	-1.0760
**fa_ac_pf**	0.25737	0.27906	0.26536	0.2587	0.28654	0.26897	0.7090	-1.5670	3.1670	-0.8260	2.0270	0.8970	-2.0270	-0.8970
**fa_ac_s1**	0.26612	0.27787	0.25767	0.25931	0.27006	0.25791	-0.6520	-1.4620	0.4030	-1.7990	1.1220	-0.1330	-1.1220	0.1330
**fa_ac_s2**	0.27224	0.29208	0.26175	0.25715	0.28087	0.26073	-0.6310	-2.3240	0.6720	-2.1900	1.2060	0.2450	-1.2060	-0.2450
**fa_ac_vp**	0.29974	0.30442	0.25798	0.27597	0.30494	0.27972	-3.0160	-2.6370	0.4000	-2.4410	3.4700	0.3880	-3.4700	-0.3880
**fa_am_ic**	0.27577	0.28263	0.25234	0.26451	0.26893	0.26909	-1.8120	-1.4730	-0.5430	-1.3070	1.4990	0.4420	-1.4990	-0.4420
**fa_am_na**	0.2534	0.28885	0.25512	0.27074	0.26015	0.27719	0.1310	-1.6620	0.4930	-0.9500	0.4040	0.5410	-0.4040	-0.5410
**fa_am_pa**	0.26908	0.29274	0.2681	0.28567	0.28257	0.28873	-0.0920	-0.6780	1.1710	-0.4020	1.0890	0.3120	-1.0890	-0.3120
**fa_am_pf**	0.25032	0.28702	0.25704	0.28093	0.26296	0.27846	0.4920	-0.3580	0.8850	-0.4040	0.3540	-0.1150	-0.3540	0.1150
**fa_am_s1**	0.27969	0.28781	0.2649	0.27841	0.26755	0.27956	-0.9770	-0.4690	-0.6110	-0.4720	0.1340	0.0540	-0.1340	-0.0540
**fa_am_s2**	0.27411	0.28503	0.26154	0.26127	0.26801	0.27042	-0.8950	-2.0350	-0.4500	-1.2350	0.4390	0.7640	-0.4390	-0.7640
**fa_am_vp**	0.29828	0.31034	0.27083	0.30918	0.3023	0.30402	-2.0620	-0.0980	0.3250	-0.7160	2.4040	-0.4860	-2.4040	0.4860
**fa_ic_na**	0.23353	0.24908	0.23828	0.23685	0.25134	0.2465	0.4190	-1.7390	1.7830	-0.3110	1.3060	1.1270	-1.3060	-1.1270
**fa_ic_pa**	0.26445	0.26088	0.25682	0.25572	0.27009	0.26097	-0.4590	-0.5230	0.3950	0.0100	0.9590	0.5990	-0.9590	-0.5990
**fa_ic_pf**	0.23824	0.243	0.24305	0.2324	0.2609	0.24322	0.4890	-1.1990	2.0960	0.0210	1.3920	1.1900	-1.3920	-1.1900
**fa_ic_s1**	0.25552	0.25975	0.23244	0.24018	0.27151	0.23613	-1.3380	-1.5100	0.9980	-2.2000	2.9560	-0.3780	-2.9560	0.3780
**fa_ic_s2**	0.2437	0.2543	0.23817	0.22926	0.24574	0.24253	-0.3930	-2.2090	0.1660	-1.0510	0.6610	1.3340	-0.6610	-1.3340
**fa_ic_vp**	0.26128	0.28072	0.27036	0.26811	0.28946	0.26414	0.5880	-1.2360	2.1110	-2.2700	1.4880	-0.3920	-1.4880	0.3920
**fa_na_pa**	0.24871	0.25595	0.24366	0.25734	0.26249	0.2562	-0.4700	0.1290	1.2540	0.0270	1.6530	-0.1190	-1.6530	0.1190
**fa_na_pf**	0.24249	0.24904	0.23731	0.23886	0.25338	0.24847	-0.4850	-0.9920	0.8930	-0.0470	1.3240	0.9180	-1.3240	-0.9180
**fa_na_s1**	0.25024	0.2535	0.2445	0.24672	0.27102	0.26504	-0.4310	-0.4690	1.2260	0.6590	1.6360	1.1940	-1.6360	-1.1940
**fa_na_s2**	0.24318	0.26222	0.23797	0.23858	0.25476	0.24349	-0.3050	-1.5740	0.6280	-1.3280	1.2340	0.3980	-1.2340	-0.3980
**fa_na_vp**	0.25131	0.27734	0.25976	0.26439	0.29497	0.26796	0.6580	-1.1300	3.9780	-0.9440	2.8730	0.3720	-2.8730	-0.3720
**fa_pa_pf**	0.2511	0.26871	0.24979	0.25974	0.26772	0.26006	-0.0800	-0.7690	1.1710	-0.6040	1.2050	0.0260	-1.2050	-0.0260
**fa_pa_s1**	0.26276	0.24996	0.25777	0.2555	0.2588	0.25644	-0.3300	0.5010	-0.3220	0.5940	0.0730	0.1060	-0.0730	-0.1060
**fa_pa_s2**	0.26637	0.28675	0.25884	0.25734	0.28111	0.2556	-0.4480	-2.5300	0.9330	-2.6250	1.4200	-0.1940	-1.4200	0.1940
**fa_pa_vp**	0.26014	0.28378	0.26073	0.25643	0.27702	0.27259	0.0480	-2.8570	1.4800	-0.9830	1.2660	1.7350	-1.2660	-1.7350
**fa_pf_s1**	0.24758	0.26005	0.23947	0.24395	0.26638	0.24123	-0.5230	-1.2220	1.2630	-2.1110	1.8870	-0.2160	-1.8870	0.2160
**fa_pf_s2**	0.23963	0.25879	0.24104	0.2358	0.25019	0.25925	0.0970	-1.2300	0.7430	0.0260	0.6250	1.3580	-0.6250	-1.3580
**fa_pf_vp**	0.26256	0.29802	0.26526	0.27207	0.26911	0.26593	0.1690	-2.2450	0.4040	-3.8950	0.2450	-0.5980	-0.2450	0.5980
**fa_s1_s2**	0.25607	0.26435	0.23248	0.23197	0.24635	0.24428	-1.4930	-2.9960	-0.6100	-1.8630	1.1360	1.5320	-1.1360	-1.5320
**fa_s1_vp**	0.28202	0.28047	0.27126	0.26433	0.29715	0.2744	-0.6080	-1.5370	1.1510	-0.7160	1.6660	1.0100	-1.6660	-1.0100
**fa_s2_vp**	0.27129	0.29126	0.26966	0.28352	0.28477	0.26693	-0.1030	-0.5630	1.0250	-2.2370	0.9680	-1.3480	-0.9680	1.3480

Supplymentary Table 5 for Figure 6. Pairwise multiple comparisons of FA values in functional connectivity among Sham, NP and ICS in each sex.
