# Supplementary material for: Sex differences in pain perception and modulation in the brain: effects of insular cortex stimulation on chronic pain relief
Source: Brain Commun. 2025 Sep 17;7(5):fcaf362. doi: 10.1093/braincomms/fcaf362 (PMC12492487; doi:10.1093/braincomms/fcaf362)
Supplement: fcaf362_Supplementary_Data [file fcaf362_supplementary_data.zip › Supplymentary Table 6 for Figure 7.pdf]

ACC-S2	Male	Female
Behavior	FA value	FA value
26.64	0.292	
25.66	0.33	
22.55	0.235	
24.45	0.28	
24.29	0.321	
22.6	0.286	
20.92	0.246	
23.92	0.248	
22	0.256	
18.71		0.243
19.27		0.264
19.84		0.243
18.28		0.304
19.81		0.278
20.11		0.306
19.61		0.366
19.68		0.351
21.51		0.34
21.67		0.258

ACC-IC	Male	Female
Behavior	FA value	FA value
8.22	0.304	
5.41	0.194	
8.43	0.207	
8.6	0.282	
8.32	0.289	
8.53	0.293	
8.39	0.28	
8.13	0.231	
7.53	0.312	
8.89		0.251
5.67		0.251
8.5		0.276
8.05		0.296
9.5		0.3
8.53		0.275
6.3		0.258
7.01		0.248
8.04		0.226
6.63		0.258

ACC-PFC	Male	Female
Behavior	FA value	FA value
8.22	0.261	
7.27	0.265	
8.4	0.299	
5.41	0.194	
8.43	0.204	
8.4	0.276	
8.32	0.303	
8.1	0.301	
7.53	0.29	
8.89		0.249
5.67		0.258
8.5		0.277
8.05		0.325
9.5		0.286
6.3		0.254
6.8		0.261
6.66		0.253
7.64		0.325
7.01		0.205

ACC-VP	Male	Female
Behavior	FA value	FA value
5.41	0.2	
8.43	0.211	
8.4	0.297	
8.6	0.295	
8.32	0.285	
7.13	0.27	
8.53	0.281	
8.13	0.221	
7.69	0.255	
5.67		0.284
8.5		0.284
8.05		0.302
9.5		0.332
8.53		0.283
6.3		0.271
6.85		0.238
6.46		0.219
6.8		0.262
6.66		0.27

Amy-IC	Male	Female
Behavior	FA value	FA value
8.22	0.285	
7.27	0.243	
5.41	0.206	
8.43	0.219	
8.4	0.272	
8.6	0.287	
8.32	0.265	
7.13	0.248	
8.53	0.269	
5.67		0.292
8.5		0.304
8.05		0.296
9.5		0.291
8.53		0.291
6.3		0.238
6.85		0.213
6.46		0.234
6.8		0.248
7.64		0.291

Nacc-PFC	Male	Female
Behavior	FA value	FA value
8.22	0.27	
7.27	0.212	
5.41	0.176	
8.43	0.19	
8.4	0.241	
8.6	0.258	
8.32	0.248	
7.13	0.252	
8.53	0.244	
8.89		0.242
5.67		0.236
8.5		0.264
8.05		0.27
9.5		0.249
8.53		0.266
6.3		0.251
6.85		0.214
6.46		0.207
6.66		0.253

ACC-PAG	Male	Female
Behavior	FA value	FA value
13.67	0.243	
15.89	0.21	
13.11	0.262	
13.44	0.267	
13.2	0.3	
15.24	0.276	
15.92	0.243	
15.76	0.307	
13.67	0.264	
14.85		0.277
14.83		0.283
12.94		0.268
14.03		0.279
14.85		0.265
14.56		0.272
12.95		0.284
13.57		0.26
13.72		0.248
12.62		0.222

ACC-PFC	Male	Female
Behavior	FA value	FA value
15.24	0.262	
15.92	0.25	
15.76	0.252	
13.67	0.292	
13.72	0.281	
13.7	0.327	
14.18	0.304	
13.82	0.265	
13.94	0.331	
11.5		0.266
14.85		0.264
14.83		0.265
12.94		0.281
14.03		0.278
14.85		0.262
14.56		0.242
12.95		0.346
12.46		0.305
14.21		0.274

ACC-S2	Male	Female
Behavior	FA value	FA value
13.67	0.238	
15.89	0.217	
13.11	0.284	
13.2	0.288	
15.24	0.265	
15.76	0.251	
13.7	0.315	
13.82	0.29	
13.94	0.306	
11.5		0.233
14.85		0.227
14.83		0.282
12.94		0.293
14.03		0.293
14.85		0.274
14.56		0.243
12.95		0.341
14.21		0.257
13.57		0.281

NAcc-S1	Male	Female
Behavior	FA value	FA value
13.67	0.223	
13.11	0.244	
15.24	0.262	
15.92	0.25	
12.94	0.324	
13.72	0.29	
13.94	0.304	
15.76	0.252	
13.67	0.298	
14.85		0.249
14.83		0.254
12.94		0.256
14.03		0.269
14.56		0.248
13.57		0.239
11		0.34
14.21		0.274
14.03		0.278
14.03		0.279

PAG-PFC	Male	Female
Behavior	FA value	FA value
13.67	0.236	
15.89	0.244	
13.44	0.289	
13.2	0.267	
15.24	0.26	
15.92	0.269	
15.76	0.261	
13.67	0.286	
13.94	0.318	
14.85		0.274
14.83		0.265
12.94		0.245
14.03		0.288
14.85		0.331
14.56		0.247
12.95		0.272
13.57		0.239
12.62		0.228
11.78		0.213

S2-VP	Male	Female
Behavior	FA value	FA value
13.67	0.251	
15.89	0.21	
15.24	0.29	
15.92	0.256	
15.76	0.221	
13.67	0.298	
14.18	0.295	
13.82	0.296	
13.94	0.344	
11.5		0.258
14.85		0.249
14.83		0.293
12.94		0.243
14.03		0.29
14.85		0.243
14.56		0.251
12.95		0.318
12.46		0.299
14.21		0.266

Supplymentary Table 6 for Figure 7. Scatter plot between FA values in functional connectivity and mechanical withdrawal threshold.
