# Supplementary figures and images for: Sex differences in pain perception and modulation in the brain: effects of insular cortex stimulation on chronic pain relief
Source: Brain Commun. 2025 Sep 17;7(5):fcaf362. doi: 10.1093/braincomms/fcaf362 (PMC12492487; doi:10.1093/braincomms/fcaf362)

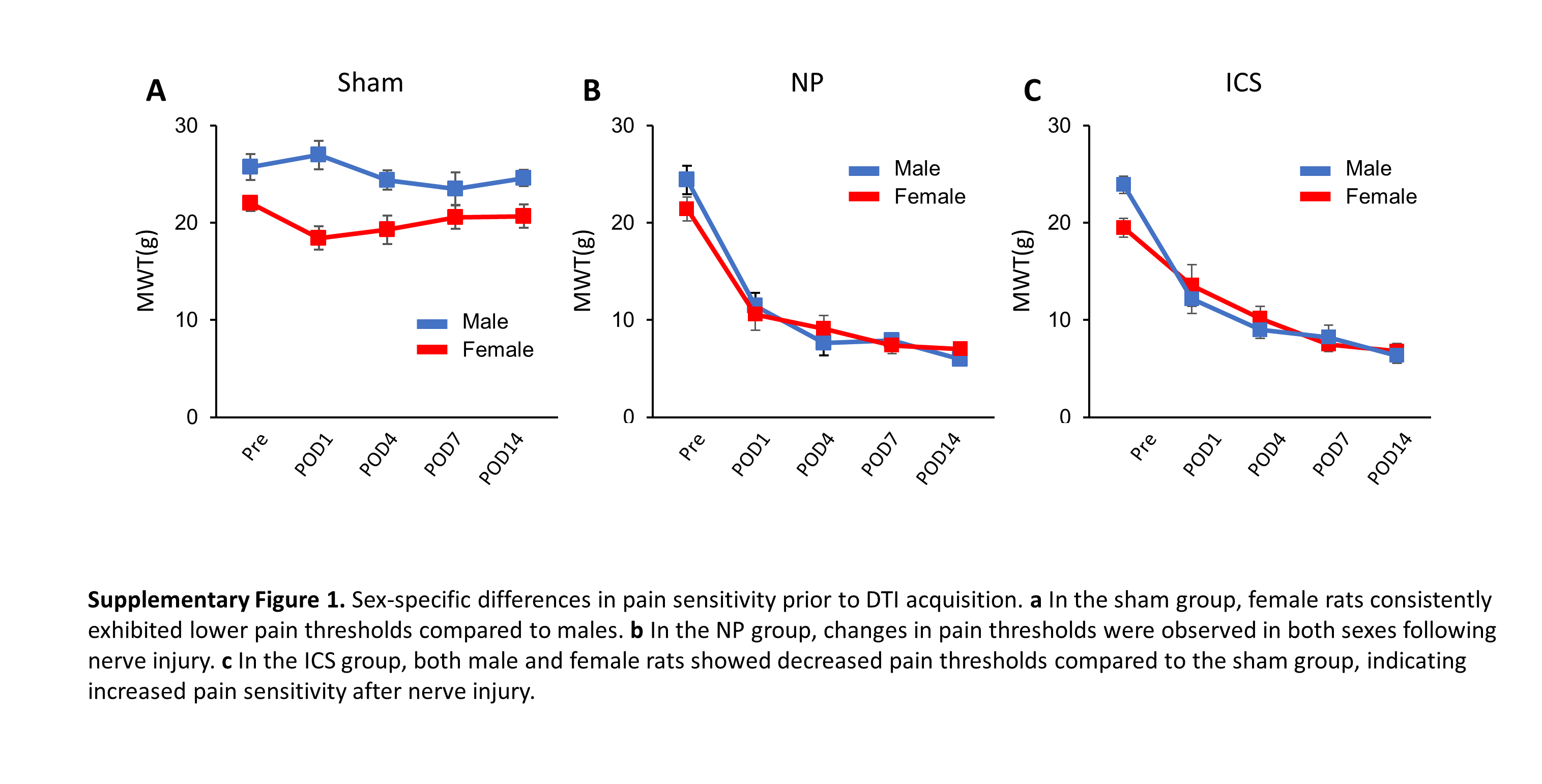

Supplement: fcaf362_Supplementary_Data [file fcaf362_supplementary_data.zip › Supplementary Figure 1.tif]
